# Pirtobrutinib inhibits wild-type and mutant Bruton’s tyrosine kinase-mediated signaling in chronic lymphocytic leukemia

**DOI:** 10.1038/s41408-022-00675-9

**Published:** 2022-05-20

**Authors:** Burcu Aslan, Gorkem Kismali, Lakesla R. Iles, Ganiraju C. Manyam, Mary L. Ayres, Lisa S. Chen, Mihai Gagea, Maria Teresa Sabrina Bertilaccio, William G. Wierda, Varsha Gandhi

**Affiliations:** 1 Department of Experimental Therapeutics, The University of Texas MD Anderson Cancer Center, Houston, TX USA; 2 Department of Bioinformatics and Computational Biology, The University of Texas MD Anderson Cancer Center, Houston, TX USA; 3 Department of Veterinary Medicine and Surgery, The University of Texas MD Anderson Cancer Center, Houston, TX USA; 4 Department of Leukemia, The University of Texas MD Anderson Cancer Center, Houston, TX USA; 5 Present Address: Department of Biochemistry, Ankara University Faculty of Veterinary Medicine, Ankara, Turkey

**Keywords:** Molecularly targeted therapy, Translational research

## Abstract

Pirtobrutinib (LOXO-305), a reversible inhibitor of Bruton’s tyrosine kinase (BTK), was designed as an alternative strategy to treat ibrutinib-resistant disease that develops due to C481 kinase domain mutations. The clinical activity of pirtobrutinib has been demonstrated in CLL, but the mechanism of action has not been investigated. We evaluated pirtobrutinib in 4 model systems: first, MEC-1, a CLL cell line overexpressing BTK^WT^, BTK^C481S^, or BTK^C481R^; second, murine models driven by MEC-1 overexpressing BTK^WT^ or BTK^C481S^; third, in vitro incubations of primary CLL cells; and finally, CLL patients during pirtobrutinib therapy (NCT03740529, ClinicalTrials.gov). Pirtobrutinib inhibited BTK activation as well as downstream signaling in MEC-1 isogenic cells overexpressing BTK^WT^, BTK^C481S^, or BTK^C481R^. In mice, overall survival was short due to aggressive disease. Pirtobrutinib treatment for 2 weeks led to reduction of spleen and liver weight in BTK^WT^ and BTK^C481S^ cells, respectively. In vitro incubations of CLL cells harboring wild-type or mutant BTK had inhibition of the BCR pathway with either ibrutinib or pirtobrutinib treatment. Pirtobrutinib therapy resulted in inhibition of BTK phosphorylation and downstream signaling initially in all cases irrespective of their BTK profile, but these effects started to revert in cases with other BCR pathway mutations such as PLCG2 or PLEKHG5. Levels of CCL3 and CCL4 in plasma were marginally higher in patients with mutated BTK; however, there was a bimodal distribution. Both chemokines were decreased at early time points and mimicked BCR pathway protein changes. Collectively, these results demonstrate that pirtobrutinib is an effective BTK inhibitor for CLL harboring wild-type or mutant BTK as observed by changes in CCL3 and CCL4 biomarkers and suggest that alterations in BCR pathway signaling are the mechanism for its clinical effects. Long-term evaluation is needed for BTK gatekeeper residue variation along with pathologic kinase substitution or mutations in other proteins in the BCR pathway.

## Introduction

In the pathophysiology of chronic lymphocytic leukemia (CLL), the B-cell receptor (BCR) pathway is an established driver of survival, proliferation, and migration of CLL lymphocytes [1, 2]. This role has been demonstrated by targeting of Bruton’s tyrosine kinase (BTK), a pivotal enzyme in the BCR signaling nexus [3], with small-molecule inhibitors, which results in prolonged disease-free survival in CLL. While several inhibitors of BTK have been tested in the clinic for B-cell malignancies including CLL, the most extensive experience has been with ibrutinib, the first BTK inhibitor in oncology.

Ibrutinib has emerged as a transformative targeted agent for B-cell malignancies in general and CLL in particular. Ibrutinib has been broadly approved for both previously untreated [4] and relapsed/refractory CLL [5]. The drug covalently and irreversibly binds to the C481 residue in the kinase domain of BTK. Ibrutinib can also bind reversibly to BTK, but with reduced potency [6]. Acalabrutinib and zanubrutinib are more selective BTK inhibitors that have been successfully used in the clinic and demonstrated clinical responses similar to those of ibrutinib, yet both had lower incidence of atrial fibrillation [7–9]. To maintain sustained inhibition of BTK and BCR signal transduction, BTK inhibitors are administered continuously, until disease progression. These therapies with covalent and irreversible binding have resulted in prolonged disease-free and overall survival in CLL; however, the persistent presence of drug has resulted in development of drug resistance due to alteration in the BCR pathway, including alteration in the binding site of ibrutinib, i.e., the C481 amino acid of BTK [10–13]. While several changes in residue C481 have been reported, the two most common alterations are cysteine to serine (C481S) or cysteine to arginine (C481R) [10, 13]. Cells harboring these mutations produce BTK that cannot be bound by the irreversible BTK inhibitors or are bound with a much lower potency (ibrutinib, acalabrutinib, and zanubrutinib), resulting in development of resistance. The majority of the work investigating these BTK inhibitors has focused on ibrutinib, as it is well-characterized and long-term follow-up studies are available [14].

To circumvent resistance, two strategies have been applied. First, irreversible BTK inhibitors have been combined with other targeted agents or anti-CD20 antibodies [15]. The second approach has been to design novel BTK inhibitors that do not bind to the C481 site, but instead inhibit the enzyme by binding reversibly in the BTK kinase pocket. Many BTK antagonists have been created using this tactic, and five agents were tested in clinical trials. These include ARQ-531 from ArQule [16], fenebrutinib (GDC-0853) from Genentech [17, 18], vecabrutinib (SNS-062) from Sunesis [19–21], CG-806 from Aptose [22], and pirtobrutinib (LOXO-305) from Loxo Oncology (now Lilly Oncology) [23, 24].

Pirtobrutinib is a novel, highly potent, third-generation noncovalent reversible BTK inhibitor. Earlier preliminary reports have shown selectivity and effectiveness of this agent in B-cell lines in vitro [25] as well as in murine models [23]. When evaluated against 370 kinases, pirtobrutinib was determined to be highly selective for BTK [24]. In a study of more than 300 patients with B-cell malignancies, drug safety and clinical outcomes were evaluated and reported [24]. In general, pirtobrutinib was well tolerated and showed efficacy in different B-cell malignancies. Particularly, in patients with CLL (*n* = 139), an overall response rate of 62% was observed. Further, pirtobrutinib was equally effective in patients who were intolerant of irreversible BTK inhibitors or developed disease that was resistant to these covalent inhibitors. Consistent with this observation, there were responses in patients who had CLL cells harboring BTK^WT^ as well as C481-mutant BTK [24].

Pirtobrutinib’s reversible binding, selectivity to BTK, high potency, desired pharmacological profile, and favorable clinical outcome in patients with WT or mutant BTK suggested potency of this agent for any BTK-driven tumor. In this study, using multiple model systems, we aimed to characterize the activity of pirtobrutinib in cells harboring either WT or mutant BTK. Previously, we established MEC-1, a CLL cell line overexpressing BTK^WT^, BTK^C481S^, or BTK^C481R^, and tested other non-covalent BTK inhibitors such as vecabrutinib [19]. Further, we developed a xenograft murine model using this cell line [26]. Using this cell line model system with diverse BTK background [19], we characterized the effect of pirtobrutinib on BCR pathway signaling. Then, we evaluated pirtobrutinib in murine models [26] that were driven by MEC-1 cell lines overexpressing either BTK^WT^ or BTK^C481S^. Thereafter, we extended in vitro incubations of primary CLL cells and compared ibrutinib with pirtobrutinib. Finally, we investigated the impact of pirtobrutinib therapy on chemokine production, considered hallmark biomarkers, and the BCR signal transduction pathway induced by diverse BTK and PLCG2 backgrounds in CLL lymphocytes obtained from patients on pirtobrutinib therapy (NCT03740529, ClinicalTrials.gov).

## Methods

### Drugs

Pirtobrutinib was provided by Loxo Oncology (Stamford, CT). Ibrutinib was purchased from Selleck Chemicals (Houston, TX). Stock solutions of both drugs were made in DMSO. Time-matched, DMSO-treated, or untreated cells were used as controls.

### CLL MEC-1 cell line and cell cultures

MEC-1 [27] was transduced to generate cell lines that stably and simultaneously overexpressed green fluorescence protein (GFP) and BTK. We generated three cell lines that overexpressed either wild-type BTK (BTK^WT^) or mutant BTK (BTK^C481R^ or BTK^C481S^) [26]. These cell lines do express endogenous BTK^WT^ protein. Cells were characterized by staining with CD23-PE Clone Tu1 (Life Technologies, Carlsbad, CA) and CD19-PE Cy7 Clone J3–119 (Beckman Coulter, Brea, CA) antibodies followed by analysis with BD Accuri C6 Plus flow cytometer (BD Biosciences, Franklin Lakes, NJ). Detailed characterization and profiling of these cells are published [26]. Prior to experiments, GFP+ cells were sorted using BD FACSAria Fusion Cell Sorter (BD Biosciences, Franklin Lakes, NJ) to obtain a pure GFP+ cell population. These cells were cultured for experiments. Some MEC-1 cells lost GFP during expansion of cultures; however, we used cell populations that were higher than 75% GFP+.

### Cell cycle, proliferation, and apoptosis assays

MEC-1 cells with BTK variants were treated with DMSO, ibrutinib, or pirtobrutinib for 24 h and were used for cell cycle, apoptosis, or proliferation assays. These procedures are described under Supplementary Methods.

### DNA and RNA synthesis assay

MEC-1 cells were treated with ibrutinib or pirtobrutinib for 1–3 days. During the last 60 min, [methyl-^3^H]thymidine (66.2 Ci/mmol) or [5,6-^3^H]uridine (27.4 Ci/mmol) were added. DNA and RNA with radioactivity were collected, and radioactivity was measured as described under Supplementary Methods.

### In vivo studies in murine model

We used modified MEC-1 cells to establish a xenograft mouse model (Supplementary Method) [26]. Diseased mice were randomly assigned to two groups: vehicle (*n* = 7 or *n* = 6 in BTK^WT^ and BTK^C481S^, respectively) and pirtobrutinib (*n* = 10, in both models). The investigators were not blinded to the experimental groups. Treatment started at day 10, and the experiment was terminated at day 26. At the endpoint, mice were euthanized, and spleens, livers, and left femurs were collected. Cells obtained from mice were stained and analyzed by flow cytometry. Mice were cared for in accordance with the guidelines by AAALAC.

### Peripheral blood collection for in vitro investigations with primary CLL cells

Patients provided written informed consent for protocols approved by the Institutional Review Board of MD Anderson Cancer Center, in accordance with the Declaration of Helsinki. Blood collection and processing are described in Supplementary Methods. Patient characteristics are listed in Table 1 and Supplementary Table 1.

**Table 1:** Characteristics of patients on pirtobrutinib trial or used for in vitro incubations.

#	Sex	Age	Rai	WBC	HGB	PLT	Neut	Lymph	B2M	IGHV	FISH	BTK status	% VAF	Prior BTKi Therapy
504*	F	60	0	14.9	12.2	143	16	81	2.6	UM	17P,P53	WT		Ibrutinib
659*	F	83	I	19.8	13	148	13	85	2.9	UM	11Q	WT		Ibrutinib
618*	F	76	0	47.5	7.7	41	0	99	9.2	UM	17p, 13Q	C481S	<5	Ibrutinib
												C481R	18	
364*	F	54	II	28.3	10	18	13	81	8.8	UM	13q14,17P,P53	C481S	NA	Ibrutinib
845*	F	64	I	13.6	11.5	104	6	91	2.7	UM	17p	C481S	30	Acalabrutinib
426	F	76	I	90.3	10.9	18	3	70	4.8	UM	13Q	C481S	<5	Acalabrutinib
												T474I	<5	
693	M	87	I	6.9	10.6	54	24	62	4.3	UM	11Q	C481S	86	Ibrutinib
321	M	79	0	120.4	12.1	195	1	95	4.1	UM	11Q	WT		Ibrutinib
561	M	56	IV	27	13.4	79	14	82	3.9	UM	13Q,13q14	C481S	20	Acalabrutinib
												T474I		
116	M	75	IV	32.2	10.5	35	3	96	3.8	UM	17p, 13Q, TP53	WT		Ibrutinib
964	M	63	IV	34.4	10.3	42	8	89	11.3	ND	17p,T12	C481S	6	Ibrutinib
536	M	84	0	18	14.5	184	28	65	4.4	UM	13Q,11q	C481S	<2	Ibrutinib
868	M	75	IV	28.1	13.7	76	19	78	2.7	ND	13Q	C481R	<5	Ibrutinib
195	M	58	IV	10.8	13.8	135	36.4	55.7	2.2	UM	Neg	C481S	13	Ibrutinib
180	M	79	IV	3.5	12.6	123	43	50	3.9	UM	11q	C481F	23	Ibrutinib
												C481S	18	
128	M	59	IV	5.7	10.6	156	68.2	22.2	4.9	UM	17p	WT		Ibrutinib
804	F	70	IV	6.1	11.7	92	28.7	64.5	4.4	UM	13Q	WT		Ibrutinib
644	M	66	0	116.3	14.2	205	13	85	3.6	UM	13Q,13q14	WT		Ibrutinib
032	M	71	0	6.9	10.7	230	63.6	26.2	4.4	M	13Q	WT		Ibrutinib
057	M	75	0	5.1	14.5	221	55.1	25.5	2.3	UM	Normal	WT		Ibrutinib
281	M	70	IV	43.1	7.1	59	1	97.0	6.2	UM	T12	C481S	63	Ibrutinib

All patients received 200 mg/d except #644 who was treated at 250 mg/d. All patients are diagnosed with CLL except #364 and #128 who were diagnosed with Richter’s syndrome prior to pirtobrutinib therapy. Samples with asterisks used for in vitro incubations.

*WBC* white blood cells, *HGB* hemoglobin, *PLT* platelet count, *Neut* neutrophil, % *Lymph* lymphocyte %, *B2M* beta 2 microglobulin, *IGHV* immunoglobulin heavy chain, *FISH* fluorescent in situ hybridization, *UM* unmutated, *M* mutated, *POS* positive, *NEG* negative, *M* male, *F* female, *13Q* 13q deletion, *T12* trisomy 12, *17p* 17p deletion, *11q* 11q deletion, *WT* BTK wild type, *VAF* variant allele frequency, *ND* not done, *NA* not available.

### Investigations with primary CLL cells during pirtobrutinib therapy

Peripheral blood samples were collected from patients (Table 1 and Supplementary Table 1) prior to therapy, i.e., cycle one, day one (C1D1), as well as 1 week (C1D8), 4 weeks or one cycle (C2D1), and three cycles (C4D1) after the start of pirtobrutinib (200 mg/d except for #644 at 250 mg/d). Plasma was collected and used for chemokine assays. Cells were isolated using Ficoll-Hypaque gradient, and cell pellets were saved for immunoblot analyses. All patients provided written informed consent to participate in the clinical trial and accompanying laboratory correlative studies. The clinical trial (identifier NCT03740529 at ClinicalTrials.gov) was approved by the Institutional Review Board of the MD Anderson Cancer Center and was conducted in accordance with the Declaration of Helsinki. Measurements of clinical laboratory endpoints are described under Supplementary Methods.

### Measurement of chemokine levels

Levels of CCL3, CCL4, CCL2, CCL5, and CCL11 in plasma were quantitated using Human ProcartaPlex Panel 1 (Thermo Fisher Scientific, Waltham, MA) as described in Supplementary Methods.

### Immunoblot analysis

MEC-1 cells or primary CLL cells were collected either after in vitro incubations with the BTK inhibitors or during pirtobrutinib therapy. Pellets were processed as described previously [26]. Immunoblots were performed with use of cellular protein extracts, and nitrocellulose membranes were probed with indicated antibodies (Supplementary Table 2) and visualized with an Odyssey Infrared Imaging System (LI-COR Biosciences, Lincoln, NE).

### Statistical analysis

Unless specified otherwise, all data were presented as mean values ± SD from at least three independent experiments. One-tailed Student *t*-tests (Fig. 2C–F) and Two-tailed Student *t*-tests were used to test the relationships between group means using GraphPad Prism software. In Figs. 4B and 5B, the variance was unequal between the groups compared. Thus, one-tailed Welch’s test was used in these figures. Wilcoxon rank sum tests were used to assess the statistical significance of differences between baseline (C1D1) and after treatment cycles in CCL chemokines. *p* values obtained after multiple tests were adjusted using Benjamini-Hochberg method. *p* values < 0.05 were considered statistically significant.

## Results

### Investigations in MEC-1 cell line with BTK variants

**Fig. 1:**
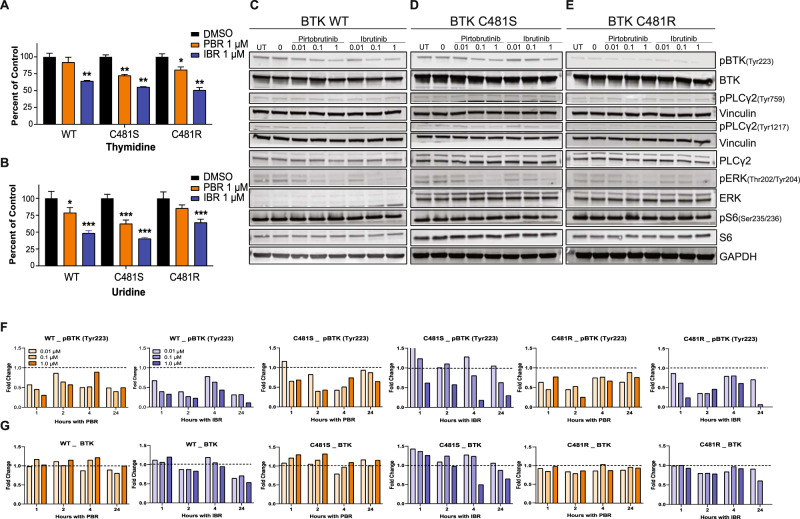
Effects of pirtobrutinib on global DNA and RNA synthesis and inhibition of proximal BCR pathway in MEC-1 cells that overexpress wild-type or mutant BTK. **A** and **B** Impact of pirtobrutinib or ibrutinib on DNA and RNA synthesis. Exponentially growing and GFP sorted MEC-1 cells overexpressing wild-type (WT), C481S, or C481R BTK were treated with 1 µM drug for 24 h. [^3^H]thymidine and [^3^H]uridine were used to determine incorporation into DNA and RNA, respectively. Data are expressed as percentage of DMSO-treated control cells. *p ≤ 0.05; **p ≤ 0.01; ***p ≤ 0.005. **C–E** Effect of pirtobrutinib or ibrutinib on BCR pathway signaling. Protein extracts were prepared from drug-treated cells and were subjected to immunoblot assays to determine levels of pBTK inhibition and downstream signaling of BTK in MEC-1 cells with BTK^WT^ (**C**), BTK^C481S^ (**D**), or BTK^C481R^ (**E**). Vinculin and GAPDH were used as the loading controls. **F** and **G** Dose- and time-dependent inhibition of BTK phosphorylation after treatment with pirtobrutinib (orange bars) or ibrutinib (blue bars) in MEC-1 cells overexpressing BTK^WT^, BTK^C481S^, and BTK^C481R^. Cells were treated at three different concentrations of drug at four different time points. Densitometry values are presented as percentage of DMSO-treated control, which is set at 1 (dashed line). PBR pirtobrutinib, IBR ibrutinib.

Phase 1 investigations demonstrated a dose-dependent increase in the steady-state level of pirtobrutinib. On day 8, 24 h after drug administration, plasma concentration at different doses ranged from 0.6 to 10 µM [24]. Ibrutinib’s peak level was 0.16 µM, and at 6 h its level was 0.06 µM [5]. Hence, we selected concentrations of 0.01, 0.1, and 1 µM for ibrutinib and pirtobrutinib. Cell death (Supplementary Fig. 1), cell cycle profiles (Supplementary Fig. 2), and proliferation index Ki67 (Supplementary Fig. 3) were not affected 24 h after incubation with pirtobrutinib or ibrutinib in MEC-1 cell lines overexpressing BTK^WT^, BTK^C481S^, or BTK^C481R^. Consistent with these data, there was a moderate decrease in thymidine incorporation with each drug. RNA synthesis was inhibited by 25–30% with pirtobrutinib and 40–50% with ibrutinib (Fig. 1A, B). Ibrutinib-mediated inhibition of nucleic acid synthesis may be due to two reasons. These cell lines express endogenous BTK^WT^, which is inhibited by both ibrutinib and pirtobrutinib. Also, ibrutinib can bind reversibly to BTK, albeit with reduced potency.

One-hour incubation of pirtobrutinib decreased phospho-BTK (Y223) and downstream phospho-ERK (T202/Y204) levels in all cell lines starting at 0.01 µM in BTK^WT^ and BTK^C481R^ and at 0.1 µM in BTK^C481S^ cells (Fig. 1C–E). Treatment with pirtobrutinib also decreased phospho-PLCγ2 (Y1217) levels at 0.01 µM in BTK^WT^ and at 0.1 µM in BTK^C481S^. This change was not detected in BTK^C481R^ cells, as phospho-PLCγ2 expression was very low in this cell line. Total and phospho-S6 protein levels remained similar in MEC-1 cells harboring BTK^WT^ or mutant BTK. Levels of total and phospho-BTK were quantitated in cells treated with 0.01, 0.1, and 1 µM ibrutinib or pirtobrutinib for different durations. In cells with BTK^WT^ treated with either drug, there was a decrease in phospho-BTK at all concentrations and times. In cells with BTK^C481S^, at all times and concentrations (except at 0.01 µM at 1 h), pirtobrutinib inhibited BTK phosphorylation. In contrast, with ibrutinib, only the highest concentration (1 µM) or longer time points inhibited BTK phosphorylation. In cells with BTK^C481R^, either drug at all concentrations was able to inhibit BTK phosphorylation (Fig. 1F). Total BTK levels generally remained similar to baseline levels after pirtobrutinib treatment while decreased with ibrutinib treatment (Fig. 1G). Phospho-ERK was reduced during the first 2 h but then increased in all cell types with either pirtobrutinib or ibrutinib, while the total ERK protein levels appeared to decrease only at higher concentrations and longer durations in BTK^WT^ and in BTK^C481S^ cell lines (Supplementary Fig. 4A, B).

### In vivo investigations in murine model with WT or mutant BTK CLL disease

**Fig. 2:**
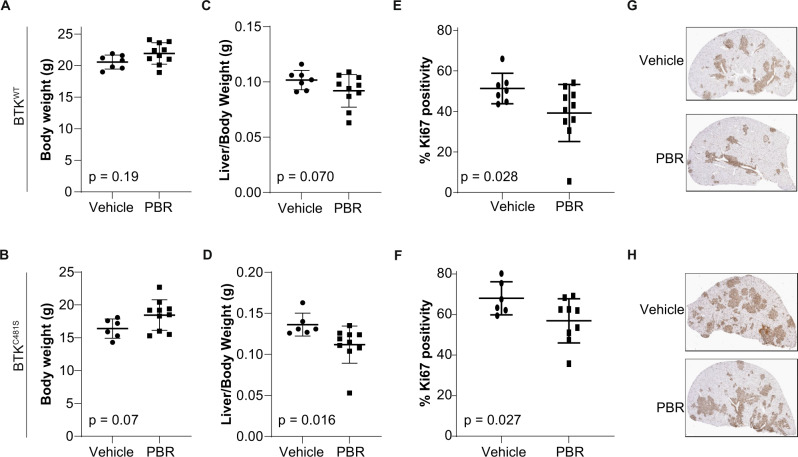
Impact of pirtobrutinib in murine model expressing MEC-1 cells harboring either BTK^WT^ or BTK^C481S^. MEC-1 cells overexpressing BTK^WT^ or mutant BTK (1 × 10^7^ cells/mouse) were injected into 8-week-old Rag2^−/−^γ_c_^−/−^ female mice and the animals were monitored daily. At day 10, mice were randomly assigned to groups: vehicle (*n* = 7 or *n* = 6 in BTK^WT^ and BTK^C481S^ models, respectively) and pirtobrutinib (*n* = 10, in both models), and treatment started. The experiment was terminated at day 26. At the endpoint, mice were euthanized, and spleens, livers, and left femurs were collected. Cells were isolated from spleens and bone marrow and stained with a monoclonal antibody against PE-labeled human CD19 Clone J3119 (Beckman Coulter) followed by flow cytometry analysis. Body weight **A** in BTK^WT^ model and **B** in BTK^C481S^ model (*P* = 0.19 and 0.07, two-tailed Student *t*-test). **C** and **D** Liver to body weight ratios of mice. Percentage Ki67 positivity in livers (*P* = 0.07 and 0.016, one-tailed Student *t*-test). **E** in BTK^WT^ model (*n* = 7 for vehicle, *n* = 10 for pirtobrutinib) (*P* = 0.028, one-tailed Student *t*-test) and **F** in BTK^C481S^ model (*n* = 6 for vehicle, *n* = 10 pirtobrutinib) (*P* = 0.027, one-tailed Student *t*-test). **G** and **H** Representative images of proliferation marker Ki-67-stained slides of livers in **G** BTK^WT^ and **H** BTK^C481S^ models before and after pirtobrutinib treatment. PBR pirtobrutinib. First row: representative images of vehicle group (top) and pirtobrutinib-treated group (lower) in BTK^WT^ model. Second row: representative images of vehicle group (top) and pirtobrutinib-treated-group (lower) in BTK^C481S^ model.

To determine the effects of pirtobrutinib treatment in an in vivo murine model of CLL, we injected either BTK^WT^ or BTK^C481S^ cells intravenously into Rag2^−/−^γ_c_^−/−^ mice previously developed and characterized by our group [26]. This murine model is very aggressive and its CLL disease develops rapidly. Pirtobrutinib treatment started at day 10 and continued for 2 weeks, as the overall survival of the mice is a maximum of 30 days. After 2 weeks of treatment with pirtobrutinib, we did not observe any loss of weight compared to vehicle-treated groups, indicating no overall toxicity in mice bearing CLL disease (Fig. 2A, B). Pirtobrutinib treatment decreased liver/body weight ratios in treatment groups compared to the vehicle groups in both BTK^WT^ and BTK^C481S^ models, but the difference was statistically significant only in BTK^C481S^ (Fig. 2C, D). Spleen/body weight ratios were also significantly decreased in the pirtobrutinib treatment group compared to the vehicle group in the BTK^WT^ model (Supplementary Fig. 5A); however, the ratios did not change in the BTK^C481S^ model (Supplementary Fig. 5B). In addition, immunohistochemistry showed that pirtobrutinib decreased the percentage of proliferative Ki67+ cells in the livers of both models (Fig. 2E, F). Representative images of Ki67-stained liver slides are provided in Fig. 2G, H. In contrast to homing of cells in the liver, there was no statistically significant difference in the number of proliferating (Ki67+) cells in spleens (Supplementary Fig. 6A, B). The total number of CD19+ cells was lower in treatment groups in the spleen (Supplementary Fig. 7A) and bone marrow (Supplementary Fig. 7C) compared to vehicle groups in the BTK^WT^ model, whereas these cells increased after pirtobrutinib treatment in the BTK^C481S^ model (Supplementary Fig. 7B and D).

### In vitro investigations in primary CLL lymphocytes treated with ibrutinib or pirtobrutinib

**Fig. 3:**
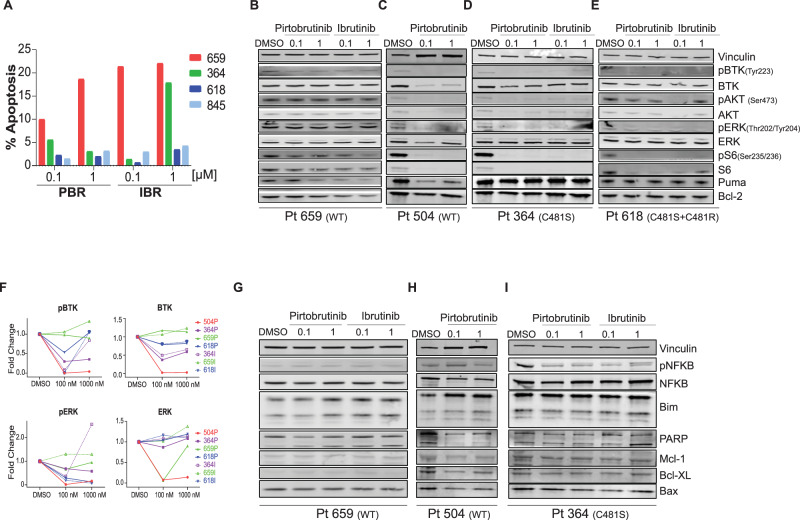
Inhibition of BCR and non-BCR pathways in CLL cells from patients with BTK^WT^ or cysteine 481 residue mutant disease after incubations with ibrutinib or pirtobrutinib in vitro. Patient blood samples were collected into Vacutainer glass green-top blood collection tubes; cells were isolated by Ficoll-Hypaque density centrifugation and were incubated with pirtobrutinib and ibrutinib at two concentrations (0.1 and 1 µM) for 24 h. **A** Apoptotic cell death in primary CLL lymphocytes of four patients. Freshly isolated cells were incubated for 24 h with indicated concentrations of pirtobrutinib. Cells were stained with annexin V–FITC and propidium iodide (PI), and apoptotic (annexin V+) cells were determined by flow cytometry. Cell death in DMSO-treated samples was subtracted from inhibitor-treated samples. **B**–**E** Effect of pirtobrutinib on BCR pathway proteins. Protein extracts were subjected to immunoblot assays to determine levels of phospho-BTK (Y223), BTK, phospho-ERK (T202/Y204), ERK, phospho-S6 (Ser235/236), and S6. **F** Graphs depict densitometry analysis for immunoblot results for phospho-BTK, BTK, phospho-ERK, ERK presented in **B**–**E**. Solid symbols represent the samples from cells treated with pirtobrutinib (unique patient ID followed by “P”), and open symbols represent samples from cells treated with ibrutinib (unique patient ID followed by “I”). **G**–**I** Effect of pirtobrutinib on non-BCR pathway proteins. Protein extracts were subjected to immunoblot assays to determine levels of phospho-NFkB, NFkB, Mcl-1, Bcl-XL, Bcl-2, Puma, Bax, and Bim. Vinculin was used as loading control. PBR pirtobrutinib, IBR ibrutinib.

Only 2–20% of patient lymphocytes with either BTK^WT^ or cysteine 481 substitutions showed induction of apoptosis after in vitro treatment with either ibrutinib or pirtobrutinib (Fig. 3A). After subtraction of the endogenous level of cell death from each sample, apoptosis rates ranged between 1.5% and 10% at 0.1 µM and between 3.2% and 18% at 1 µM of pirtobrutinib treatment. Among the BCR pathway signaling proteins (Fig. 3B–E), phospho-BTK (Tyr223) was inhibited in all samples independent of BTK mutation status. Phospho-AKT and phospho-ERK levels varied between patients but did not correlate with any specific BTK status. Our densitometry analysis showed that treatment with 0.1 µM ibrutinib or pirtobrutinib decreased phospho-BTK and phospho-ERK levels in all samples except ibrutinib-treated cells in patient 659. In addition, total BTK levels decreased in all patients except patient 659. Total ERK levels decreased in patients 504, 659, and 364 after 0.1 µM pirtobrutinib treatment (Fig. 3F). Phospho-S6 (Ser235/236) and S6 levels were abrogated after pirtobrutinib treatment in all samples. There were no apparent differences in response between the two BTK inhibitors during in vitro incubations. Among non-BCR proteins, Bim protein levels increased following treatment with either ibrutinib or pirtobrutinib (Fig. 3G–I).

### Changes in plasma cytokine levels in patients during pirtobrutinib therapy

**Fig. 4:**
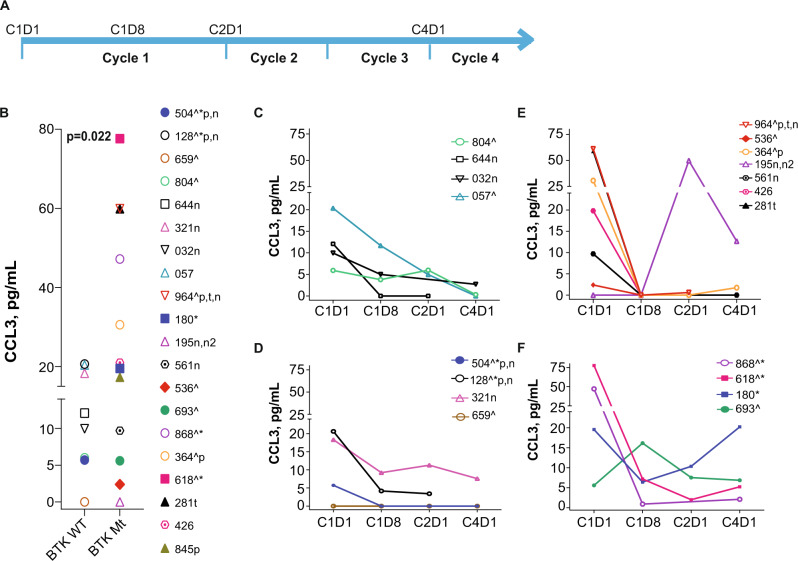
Inhibition of CCL3 chemokine production during pirtobrutinib therapy in CLL patients previously treated with irreversible BTK inhibitors. Peripheral blood samples were collected prior to therapy (C1D1) and 1 week (C1D8), 4 weeks or one cycle (C2D1), and three cycles (C4D1) after the start of pirtobrutinib. **A** Schema depicts the time points that samples were collected in the study. This study is registered at ClinicalTrials.gov (identifier NCT03740529). Plasma was collected at indicated time points and used for chemokine assays. CCL3 (Mip-1α) **B–F** levels were quantitated using Luminex XMap Technology as described under Supplementary Methods. **B** CCL3 levels at baseline (C1D1) in plasma of patients with either WT BTK or mutant BTK (*P* = 0.022, one-tailed Welch’s *t*-test) (**C**) and (**D**) Changes in CCL3 levels during therapy in patients harboring WT BTK CLL cells without PLCG2 or PLEKHG5 mutations (**C**) or in patients harboring WT BTK CLL cells with PLCG2 and/or PLEKHG5 mutations (**D**). **E** and **F** Changes in CCL3 levels during therapy in patients harboring mutant BTK CLL cells without PLCG2 or PLEKHG5 mutations (**E**) or in patients harboring WT BTK CLL cells with PLCG2 and/or PLEKHG5 mutations (**F**). Study included patients with WT BTK (*n* = 8) and mutant BTK (*n* = 12). BTK double kinase mutants are depicted by solid squares (patients 618 [BTK^C481S+C481R^] and 180 [BTK^C481F+C481S^]). BTK kinase domain as well as gatekeeper mutants are indicated by a hexagon with a dot (patients 426 [BTK^C481S+T474^] and 561 [BTK^C481S+T474^]). Patient 845 received pirtobrutinib and venetoclax combination and was not included in the time course; patients 364 and 128 had Richter transformation. Patients’ additional mutations are indicated in the key by a caret (TP53), navy blue color (BCL2), n (NOTCH1), n2 (NOTCH2), and asterisk (PLCG2), p indicates 17pdel and t indicates trisomy 12.

Plasma samples from patients on pirtobrutinib therapy were collected at 4 time points as indicated in Fig. 4A. The baseline plasma levels of CCL3 (Fig. 4B) showed heterogeneity among patients and were marginally higher in patients harboring BTK alterations (*n* = 12) compared to those harboring BTK^WT^ (*n* = 8); however, there appeared to be a bimodal distribution. Those with >20 pg/mL included both patients with trisomy 12 and one patient with Richter’s transformation. Levels of these cytokines were not related to lymphocyte, white blood cell, lactate dehydrogenase level or age but showed a significant direct linear relationship with IgM and B2M levels (Supplementary Fig. 8). In all patients analyzed, as an aggregate, plasma levels of CCL3 were inhibited as early as 1 week after the start of pirtobrutinib (C1D8) and remained low during three cycles of therapy (Supplementary Fig. 9).

We next assessed changes in CCL3 levels in patients with diverse BCR pathway backgrounds. For patients with BTK^WT^ with or without PLCG2 or PLEKHG5 mutations, there was a slow and steady decline in CCL3 levels; however, in two patients with these aberrations, high levels were maintained (Fig. 4C, D). For all samples with BTK alterations (including C481 and T474 double mutants) without PLCG2 or PLEKHG5 anomalies, CCL3 sharply declined within the first 8 days and generally remained low except in one patient (195) who had both NOTCH1/2 mutations (Fig. 4E). BTK single or double kinase alterations along with PLCG2 and PLEKHG5 abnormalities showed heterogenous responses, and in some cases, CCL3 levels increased (Fig. 4E).

**Fig. 5:**
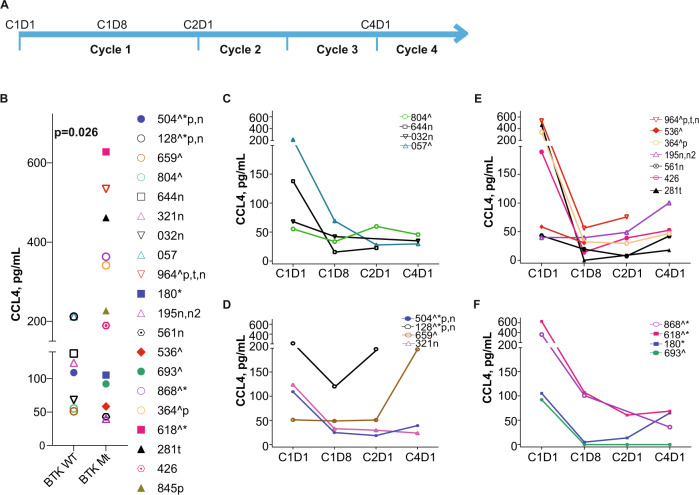
Inhibition of CCL4 chemokine production during pirtobrutinib therapy in CLL patients previously treated with irreversible BTK inhibitors. Peripheral blood samples were collected prior to therapy (C1D1) and 1 week (C1D8), 4 weeks or one cycle (C2D1), and three cycles (C4D1) after the start of pirtobrutinib. **A** Schema depicts the time points that samples were collected in the study. This study is registered at ClinicalTrials.gov (identifier NCT03740529). Plasma was collected at indicated time points and used for chemokine assays. CCL4 (Mip-1β) **B**–**F** levels were quantitated using Luminex XMap Technology as described under Supplementary Methods. **B** CCL4 levels at baseline (C1D1) in plasma of patients with either WT BTK or mutant BTK. (*P* = 0.026, One-tailed Welch’s *t*-test). **C** and **D** Changes in CCL4 levels during therapy in patients harboring WT-BTK CLL cells without PLCG2 or PLEKHG5 mutations (**C**) or in patients harboring WT BTK CLL cells with PLCG2 and/or PLEKHG5 mutations (**D**). **E** and **F** Changes in CCL4 levels during therapy in patients harboring mutant BTK CLL cells without PLCG2 or PLEKHG5 mutations (**E**) or in patients harboring WT BTK CLL cells with PLCG2 and/or PLEKHG5 mutations (**F**). Study included patients with WT BTK (*n* = 8) and mutant BTK (*n* = 12). BTK double kinase mutants are depicted by solid squares (patients 618 [BTK^C481S+C481R^] and 180 [BTK^C481F+C481S^]). BTK kinase domain as well as gatekeeper mutants are indicated by a hexagon with a dot (patients 426 [BTK^C481S+T474^] and 561 [BTK^C481S+T474^]). Patient 845 received pirtobrutinib and venetoclax combination and was not included in the time course; patients 364 and 128 had Richter transformation. Patients’ additional mutations are indicated in the key by a caret (TP53), navy blue color (BCL2), n (NOTCH1), n2 (NOTCH2), and asterisk (PLCG2); p indicates 17pdel and t indicates trisomy 12.

The basal levels of CCL4 (Fig. 5B), while much higher, mimicked CCL3 profiles and were marginally greater in patients harboring BTK mutations. Again, levels of these cytokines were not related to lymphocyte, white blood cell, or lactate dehydrogenase level or age but showed a significant direct linear relationship with IgM and B2M levels (Supplementary Fig. 10). In all patients analyzed, as an aggregate, plasma levels of CCL4 were inhibited as early as a week after pirtobrutinib treatment (C1D8) and remained low during three cycles of therapy (Supplementary Fig. 11).

We next assessed changes in CCL4 levels in patients with diverse BCR pathway backgrounds. For patients with WT BTK with or without PLCG2 or PLEKHG5 alterations, there was a slow and steady decline in CCL4 levels; however, these levels started to increase in patients with PLCG2 or PLEKHG5 mutations (Fig. 5C, D). For all samples with BTK anomalies (including C481 and T474 double mutants) without PLCG2 or PLEKHG5 anomalies, CCL4 declined within the first cycle of treatment (Fig. 5E) but then increased in most patients (Fig. 5E, F).

The highest baseline CCL2 levels were observed in the patients who had BTK, p53, and PLCG2 mutations simultaneously (patients #618 and #868) (Supplementary Fig. 12A, B). CCL2 levels during therapy either remained steady or decreased at C1D8 and/or C2D1. CCL5 levels were also similar across all time points and all patients except one who showed an increase after C1D8 (Supplementary Fig. 12C, D). CCL11 levels increased in most of the patients at either C1D8 or C2D1 regardless of their mutation status. However, we observed a dramatic decrease in two patients who had PLCG2 alterations (patients 618 and 180) (Supplementary Fig. 12E, F). In contrast to CCL3 and CCL4, aggregate levels of CCL2, CCL5, and CCL11 were not significantly changed from baseline values after treatment with pirtobrutinib.

### Investigations in CLL lymphocytes from patients during pirtobrutinib therapy

**Fig. 6:**
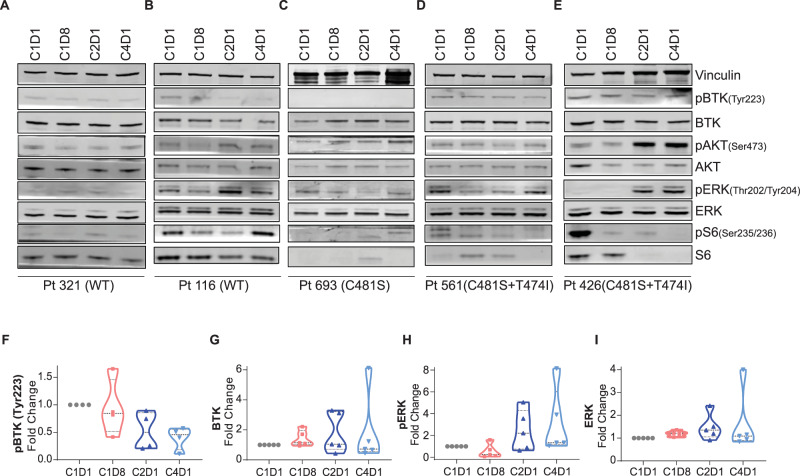
Inhibition of BCR pathway during pirtobrutinib therapy in CLL patients previously treated with irreversible BTK inhibitors. Peripheral blood samples were collected prior to therapy (C1D1) and 1 week (C1D8), 4 weeks or one cycle (C2D1), and three cycles (C4D1) after the start of pirtobrutinib. Samples were processed and cells were isolated by Ficoll-Hypaque density centrifugation. **A**–**E** Effect of pirtobrutinib on BCR pathway proteins. Protein extracts were subjected to immunoblot assays to determine levels of phospho-BTK (Y223), BTK, phospho-AKT, AKT, phospho-ERK (T202/Y204), ERK, phospho-S6 (Ser235/236), and S6. Vinculin was used as loading control. **F**–**I** Violin plots depicting changes in phospho-BTK (**F**), total BTK (**G**), phospho-ERK (**H**), and total ERK (**I**) during therapy. Proteins were quantitated, normalized with vinculin, and presented as fold change to baseline (C1D1) value.

**Fig. 7:**
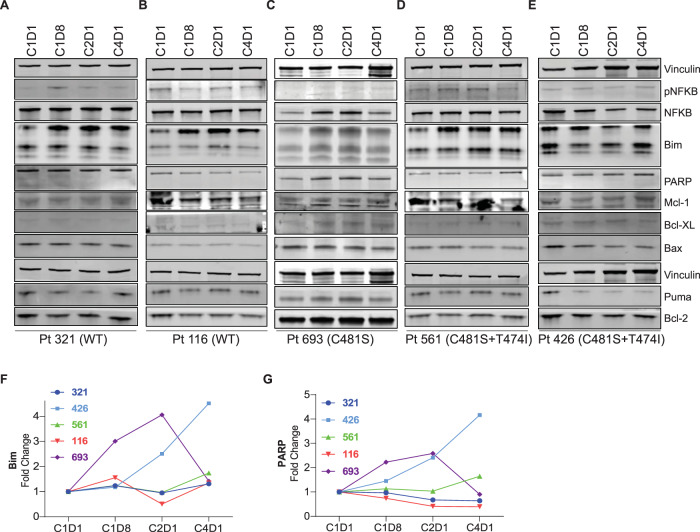
Alterations in non-BCR proteins during pirtobrutinib therapy in CLL patients with ibrutinib-resistant disease. Patient blood samples were collected (as in Fig. 6), and cells were isolated by Ficoll-Hypaque density centrifugation. **A**–**E** Protein extracts were subjected to immunoblot assays to determine levels of phospho-NFkB, NFkB, Mcl-1, Bcl-XL, Bcl-2, Bax, Puma, PARP, and Bim. Vinculin was used as loading control. **F**, **G** Graphs depict densitometry analysis for immunoblots results of Bim (**F**) and PARP (**G**). Each patient is represented by a colored symbol and a unique ID.

CLL patients enrolled in this study had diverse BTK and BCR pathway profiles. Immunoblot and densitometry analysis showed that BTK phosphorylation was decreased and remained inhibited in CLL cells of all patients irrespective of their BTK mutation status (Fig. 6A–F), while total BTK protein levels remained unchanged. Phospho-AKT levels were initially lowered but then increased following one cycle of therapy in all patient samples tested. Phospho-ERK levels also appeared to increase following one or two cycles of therapy, especially in CLL cells with mutant BTK (Fig. 6A–E and H, I). Phospho-AKT and phospho-ERK increases were evident in patient 116 (PLEKHG5 mutation), patients 561 and 426 (BTK kinase and gatekeeper residue variation), and patient 426 (NFKBIE and MAPK1 anomalies). Phospho-S6 levels demonstrated heterogeneous responses and generally decreased (Fig. 6A–E). Among Bcl-2 family members, we observed an induction of Bim protein levels, albeit with heterogeneity during therapy (Fig. 7A–E) that was confirmed by densitometric analysis (Fig. 7F). Our analysis also showed that PARP was induced following 1 week and enhanced up to 2-fold after one cycle of pirtobrutinib therapy in two patients (426 and 561) who had double BTK substitutions (C481S and T474) (Fig. 7G). These data suggest that pirtobrutinib treatment initially inhibits the BCR signaling pathway in CLL patients irrespective of their BTK status; however, this inhibition was reversed after one cycle of therapy.

## Discussion

Several novel reversible BTK inhibitors have been designed to treat disease refractory to ibrutinib owing to mutation in the C481 kinase domain. Pirtobrutinib previously appeared to result in a high (>60%) overall response rate for previously heavily treated CLL and small lymphocytic lymphoma. Additionally, similar responses were observed in patients who were intolerant or resistant to other covalent inhibitors, including patients with C481 residue variants. Pirtobrutinib was also effective in patients with Waldenstrom macroglobulinemia, mantle cell lymphoma [24, 28], and Richter’s transformation [29]. Overall, these previous results suggest efficacy of this new drug for B-cell malignancies in general and CLL in particular. However, changes in molecular targets, impact on the B-cell receptor proximal pathway, and other distal events have not been reported yet; this preclinical and clinical characterization is the focus of the present report.

Using four different model systems or primary malignant cells, we evaluated pirtobrutinib-mediated inhibition of the BCR signaling cascade and biological effects. The MEC-1 cell line is considered to have low BCR signaling and shows very limited sensitivity to ibrutinib [30]. However, we previously showed that overexpression of wild-type or specific mutant clones such as BTK^C481S^ and BTK^C481R^ resulted in activated BCR signaling. Total and phospho-BTK or phospho-ERK levels were induced upon IgM stimulation [26]. We also showed that ibrutinib treatment resulted in dramatic decrease in phospho-BTK and phospho-ERK levels in MEC-1 cells that overexpress wild-type BTK [26]. In another study, we showed that non-covalent BTK inhibitors such as vecabrutinib inhibited BCR signal transduction in these cell line model systems [19]. Collectively, these data established BCR signaling in MEC-1 cells with transduced BTK. Our current in vitro data clearly demonstrate pirtobrutinib-mediated inhibition of the BCR signaling axis in cell line model systems expressing high levels of BTK^WT^, BTK^C481S^, and BTK^C481R^ (Fig. 1).

To mimic ibrutinib resistance in vivo, we developed a xenograft model and intravenously injected transduced MEC-1 cells overexpressing wild-type or mutant BTK. In this model system, the disease develops rapidly and progresses aggressively. Thus, the overall survival of mice does not exceed 30 days, leading to a limited window for duration of treatment. Survival studies also have been done in Eµ-TCL1 mice with irreversible BTK inhibitors such as ARQ-531 [16] or vecabrutinib [21], but these models do not have BTK mutations. The absence of non-tumor immune cells in our transplanted mice is another limitation of our system. In addition, it appears that in this model, the liver is the primary site of disease development, rather than the spleen. The liver is where we observed changes after pirtobrutinib treatment (Fig. 2C, D) and changes in the proliferation index of MEC-1 cells, harboring either wild-type or mutant BTK (Fig. 2E–H). Although our in vivo data are not solely sufficient to draw conclusions about the resistance mechanism, they support the results we observed in three other model systems.

When extended to primary CLL lymphocytes, this inhibitory activity was confirmed in CLL cells harboring BTK^WT^ or mutant BTK during in vitro investigation (Fig. 3). Next, to demonstrate that pirtobrutinib had similar effect on the BCR signal transduction pathway, we analyzed data at different time points after the start of 200 mg/day of pirtobrutinib (Figs. 6 and 7). Data during therapy further validated the impact of pirtobrutinib on the BCR pathway as early as a week after the start of therapy.

As in the parent study [24], the patients in our study, who were participating in the trial, had been treated previously with BTK inhibitors. CLL cells from these patients showed decreased BCR pathway signaling after 1 week or one cycle of pirtobrutinib (Fig. 6). This inhibition was observed irrespective of BTK status, wild-type or mutated, in malignant lymphocytes.

A hallmark of covalent as well as non-covalent BTK inhibitors’ action is a decrease in CCL3 and CCL4 during therapy. The molecular interaction between CLL cells and their microenvironment is prominent, as it affects cell survival and proliferation. Upon BCR activation, CLL cells secrete chemokines such as CCL3 and CCL4 for the recruitment of immune cells [31]. CLL patients show increased plasma levels of CCL3 and CCL4 [32], and CCL3 is associated with shorter time to disease progression in CLL [33]. It has been also reported that elevated plasma CCL3 and CCL4 levels in CLL patients decrease rapidly upon treatment with ibrutinib [34, 35]. In our study, there was a similar decline in these chemokines in plasma samples of patients with BTK^WT^, kinase domain mutants, or double variants with gatekeeper mutations in BTK (Figs. 4 and 5). Once again, in most cases, these declines were within the first cycle. At later time points, there were increases in chemokine levels in patients with kinase and gatekeeper residue variations (Fig. 5E) or with PLCG2 or PLEKHG5 mutations (Fig. 5F). This result may reflect clonal evolution of respective mutated clones. While immunoblot data regarding AKT and ERK phosphorylation mirrored CCL4 changes, additional longitudinal samples need to be analyzed to determine response or disease progression in these patients.

These data suggest that pirtobrutinib’s pharmacokinetic/pharmacodynamic profile during therapy was sufficient to result in initial mechanistic inhibition of BCR pathway in CLL cells with a decline in CCL3/CCL4 biomarkers among diverse BTK backgrounds. The recommended phase 2 dose of pirtobrutinib is 200 mg/day. At this dose, the peak level in plasma, which is generally attained within the first 2 h after administration, is ~15 µM [24]. Pirtobrutinib has a relatively long half-life of 20 h with the steady-state concentration of ~8 µM (measured at 24 h on day 8). On the other hand, at the recommended and prescribed dose of 420 mg/day, ibrutinib’s peak plasma level achieved at 2 h was 0.16 µM. This peak was eliminated with an initial half-life of 2 h and a terminal elimination rate of ~8 h. At 6 h after drug intake, a plasma level of only 60 nM was observed [5]. These data clearly demonstrate that, compared with ibrutinib, pirtobrutinib has a better, and favorable, pharmacokinetic profile with an almost 90-fold higher peak level and 2.5-fold better retention of the drug in human plasma.

However, another factor in this equation manifested because the two drugs work differently in BTK^WT^ disease. Ibrutinib is an irreversible and covalent inhibitor. Once it binds to the kinase, it inactivates or kills the enzyme. Further, after one cycle of full-dose ibrutinib, the transcript and protein levels of BTK are decreased [36, 37], and a lower dose of ibrutinib is sufficient for pharmacodynamic effect measured as target occupancy [37]. Pirtobrutinib, on the other hand, is a reversible inhibitor and does not irreversibly inactivate (kill) the BTK enzyme. Hence, levels of BTK do not decline with this inhibitor. Rather a continuous tonic presence is needed to bind and inhibit the target, BTK. The long half-life of pirtobrutinib and a favorable binding efficiency to BTK may make this reversible BTK inhibitor an effective drug.

For pathogenic BTK (mutated in the cysteine 481 residue), a direct comparison of ibrutinib and pirtobrutinib could be done because, in this scenario, ibrutinib does not bind covalently but rather inhibits the variant kinase by irreversible binding [6]. Under these circumstances (C481-mutated BTK), pirtobrutinib will have a favorable pharmacodynamic effect on the target. It is currently challenging to compare these two agents for other variants of BTK mutation, such as gatekeeper residue (T474) variations [38]. Taken together, kinome profiling data of ibrutinib and pirtobrutinib against BTK^WT^ and variants, and pharmacokinetics data of both drugs at the prescribed dose and schedule, suggest superiority of pirtobrutinib over ibrutinib for patients harboring any variant of C481-mutated BTK enzyme. However, further clinical studies are needed to confirm the benefit.

Additionally, this sensitivity could be explained by the affinity and inhibitory potency of pirtobrutinib. BTK^WT^ was inhibited by ibrutinib with an IC_50_ of 0.5 nM [6] and by pirtobrutinib at <5 nM [25]. For cysteine 481 substitutions (C481S, C481T, C481R), the IC_50_ of pirtobrutinib was ≤10 nM while that of ibrutinib was 100 nM for C481S and inactive for the other two mutants [23]. Pirtobrutinib targeted BTK^WT^ with maximum selectivity, followed by mutant BTK^C481S^. In contrast, ibrutinib inhibited 10 kinases at a similar concentration to that of BTK or at 2- to 21-fold higher concentrations [6]. Some of the off-target effects of ibrutinib were attributed to inhibition of several other kinases in addition to BTK. On the other hand, inhibition of MEK1/2 by pirtobrutinib may help in its activity against BTK-mutant CLL[25].

In conclusion, pirtobrutinib appears to be an effective agent for lowering BCR-mediated signaling in cell lines as well as primary cells, including during therapy. Importantly, this drug inhibits signal transduction initiated either from wild-type BTK or kinase domain-mutant BTK. The initial inhibition of signal transduction emitting from the BCR pathway indicates successful inhibition of the BCR axis. Development of a BCR-independent clone or expansion of clones mutated in the molecules downstream of BTK needs to be evaluated along with combination strategies with pirtobrutinib.

## Supplementary information

Supplemental Methods

Supplementary Figures

Supplementary Figure Legends

Supplementary Tables
