# Supplementary material for: Pirtobrutinib inhibits wild-type and mutant Bruton’s tyrosine kinase-mediated signaling in chronic lymphocytic leukemia
Source: Blood Cancer J. 2022 May 20;12(5):80. doi: 10.1038/s41408-022-00675-9 (PMC9123190; doi:10.1038/s41408-022-00675-9)
Supplement: Supplementary file 1 — Supplemental Methods [file 41408_2022_675_MOESM1_ESM.docx]

**Supplemental Methods**

***CLL MEC-1 cell line and cell cultures***

All cell lines were routinely screened for *Mycoplasma* species using the MycoAlert Mycoplasma Detection Kit (Lonza, Morristown, NJ). The MEC-1 parental cell line was authenticated by the Cytogenetics and Cell Authentication Core Facility of MD Anderson Cancer Center. GFP-labeled MEC-1 cells were sorted using BD FACSAria Fusion Cell Sorter (BD Biosciences, Franklin Lakes, NJ) for the enrichment of GFP+ cells. We observed that GFP positivity rate in MEC-1 cells decreased during expansion of cultures to achieve the desired number of cells for further experiments. Therefore, we determined the percentage of GFP positivity before each experiment and aimed to use cell populations that were higher than 75% GFP+ in all experiments.

***Cell cycle, proliferation, and apoptosis assays***

For cell cycle, MEC-1 cells with BTK variants were either treated with DMSO, ibrutinib, or pirtobrutinib for 24 hours and were used for cell cycle, apoptosis, or proliferation assays. For cell cycle analyses, cells were washed in phosphate buffered saline (PBS) and fixed overnight in 70% ethanol. Cells were then centrifuged and reconstituted in PBS with 50 μg/mL propidium iodide (PI). PI fluorescence was assessed by flow cytometry (BD Accuri C6, BD Biosciences), and the percentage of cells in each cell cycle phase was analyzed with use of BD Accuri C6 software (BD Biosciences, Franklin Lakes, NJ).

For apoptosis, at the indicated time points, cells were stained with PE-labeled-annexin V (BD Biosciences) and percentage of apoptotic (AnnexinV+) cells were quantitated using BD Accuri C6 plus flow cytometer. For proliferation, cells were stained with PE-labeled-Ki67 antibody (BD Biosciences). The proliferative cells (Ki67+) were determined by flow cytometry (BD Accuri C6, BD Biosciences).

***DNA and RNA synthesis assay***

MEC-1 cells were treated with ibrutinib or pirtobrutinib for 1 to 3 days. During the last 60 minutes, [methyl-^3^H]thymidine (66.2 Ci/mmol) or [5,6-^3^H]uridine (27.4 Ci/mmol) were added. Cells were collected on Cytiva Whatman GF/C glass microfiber filters (Marlborough, MA) pre-wetted with 1% sodium pyrophosphate, washed twice with 5 ml of 0.4N perchloric acid, 70% ethanol, and 100% ethanol, and dried overnight. Filter collected DNA and RNA with radioactive precursors were counted using Tri-Carb 4910 TR liquid scintillation analyzer (Perkin Elmer, Waltham, MA). Radioactivity was expressed as percent of control (DMSO treated).

**In vivo *studies in murine model***

We used modified MEC-1 cells to establish xenograft mouse model; detailed description of this model is recently published (1). Briefly, 1 × 10^7^ MEC-1 cells (BTK^WT^ or BTK^C481S^) were injected intravenously into 8-week-old female Rag2^−/−^γ_c_^−/−^ mice that were monitored daily. Mice were randomly assigned to two groups: Vehicle (n=7 or n=6 in BTK^WT^ and BTK^C481S^ models, respectively) and Pirtobrutinib (n=10, in both models). Treatment started at day 10 and the experiment was terminated at day 26. At endpoint, mice were euthanized and spleens, livers, and left femurs were collected. Cells were isolated from spleen and bone marrow, stained with a PE labeled monoclonal antibody against human CD19 Clone J3119 (Beckman Coulter), and analyzed by flow cytometry. Institutional Animal Care and Use Committee approved and supervised all animal studies. Mice were cared for in accordance with the guidelines by the American Association for Accreditation of Laboratory Animal Care (AAALAC) and the US-PHS Policy on Humane Care and Use of Laboratory Animals.

***Peripheral Blood Collection for in vitro investigations with primary CLL cells***

Peripheral blood samples were collected into Vacutainer glass green top blood collection tubes (Becton Dickinson, [Franklin Lakes, NJ](https://www.google.com/search?sxsrf=ALeKk0332luipS0iKOI9LSaBEqHif-H1ew:1618950565680&q=Franklin+Lakes,+New+Jersey&stick=H4sIAAAAAAAAAOPgE-LUz9U3MCozKDZV4gAxK7ILK7S0spOt9POL0hPzMqsSSzLz81A4VhmpiSmFpYlFJalFxYtYpdyKEvOyczLzFHwSs1OLdRT8UssVvIBSqZU7WBkBrKKs_2MAAAA&sa=X&ved=2ahUKEwjT4YDr1I3wAhWVds0KHcZeBX0QmxMoATAregQITxAD)) and PBMCs were isolated by Ficoll-Hypaque density centrifugation (Atlanta Biologicals, Norcross, GA). The cells were washed twice with PBS, and the number of cells and cell volume were determined using a Coulter Channelyzer (Beckman Coulter, Brea, CA) and were suspended in RPMI-1640 medium with 10% human serum (Sigma Aldrich, St. Louis, MO), incubated for 24 hours with either pirtobrutinib or ibrutinib.

***Clinical laboratory end points***

**Patient clinical characteristics were analyzed at MD Anderson Cancer Center.** Determination of *IGHV* gene mutation status and ZAP-70 analyses were performed as described (2). Conventional karyotyping and fluorescence in situ hybridization (FISH) were performed by the clinical cytogenetics laboratory, Department of Hematopathology at MD Anderson Cancer Center. Detailed methodology for the FISH assay was published(3). All patient samples were analyzed genomically using the CLL panel.

***Measurement of chemokine levels***

Plasma samples were collected during therapy. Levels of CCL3, CCL4, CCL2, CCL5 and CCL11 were quantitated using Cytokine/Chemokine/Growth Factor 45-Plex Human Procartaplex Panel 1 (Thermo Fisher Scientific, Waltham, MA). Chemokine levels were quantitated using Luminex XMap Technology. The quantitation and analyses were performed at Orion Core Facility at MD Anderson Cancer Center. The results are presented as the means of technical triplicates in pg/mL.

**REFERENCES**

1. Aslan B, Kismali G, Chen LS, Iles LR, Mahendra M, Peoples M, et al. Development and characterization of prototypes for in vitro and in vivo mouse models of ibrutinib-resistant CLL. Blood Advances. 2021;5(16):3134-46.

2. Rassenti LZ, Huynh L, Toy TL, Chen L, Keating MJ, Gribben JG, et al. ZAP-70 compared with immunoglobulin heavy-chain gene mutation status as a predictor of disease progression in chronic lymphocytic leukemia. New England Journal of Medicine. 2004;351(9):893-901.

3. Glassman AB, Hayes KJ. The value of fluorescence in situ hybridization in the diagnosis and prognosis of chronic lymphocytic leukemia. Cancer genetics and cytogenetics. 2005;158(1):88-91.
