# Supplementary material for: Pirtobrutinib inhibits wild-type and mutant Bruton’s tyrosine kinase-mediated signaling in chronic lymphocytic leukemia
Source: Blood Cancer J. 2022 May 20;12(5):80. doi: 10.1038/s41408-022-00675-9 (PMC9123190; doi:10.1038/s41408-022-00675-9)
Supplement: Supplementary file 2 — Supplementary Figures [file 41408_2022_675_MOESM2_ESM.pptx]

## Slide 1
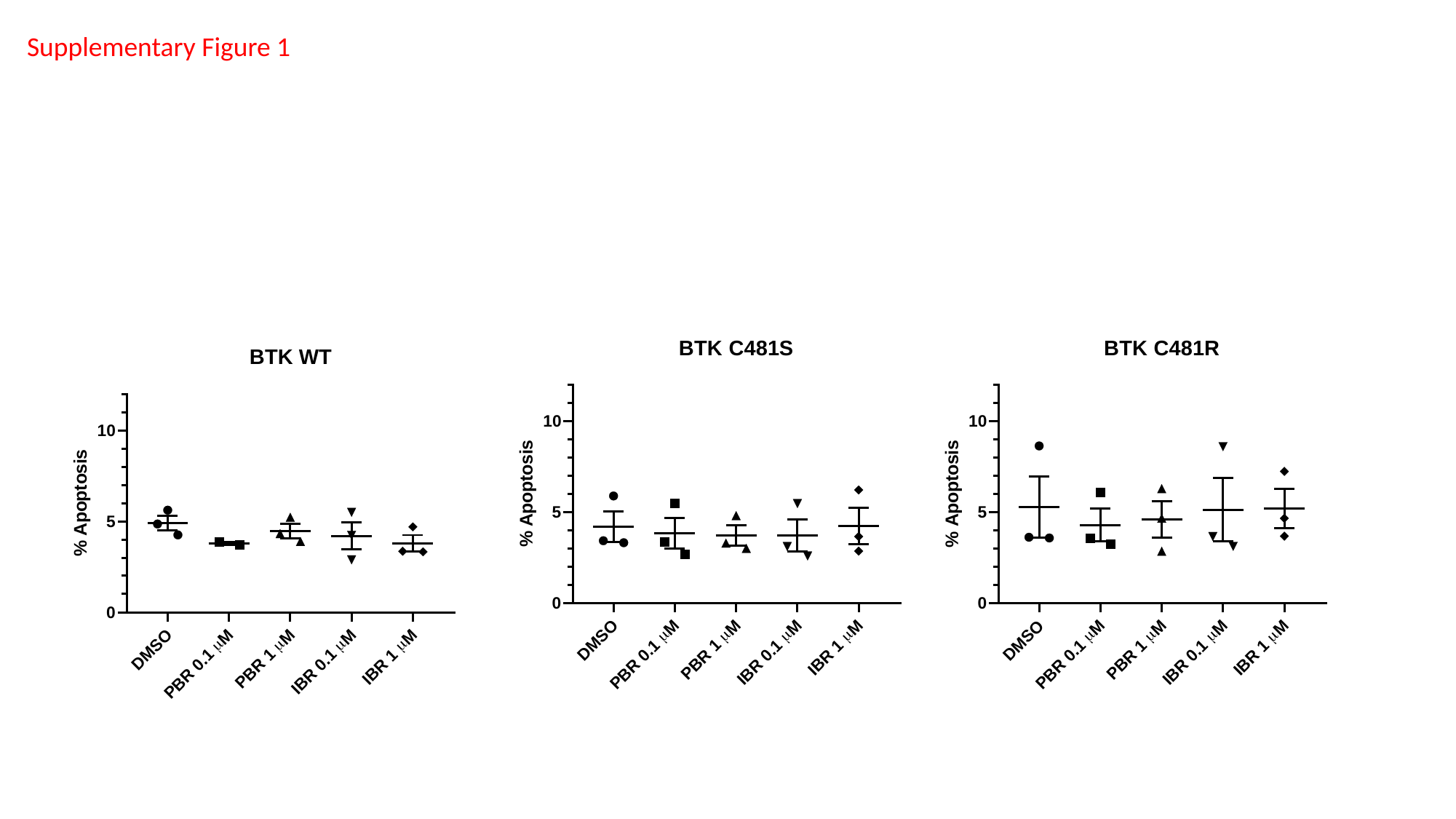

Supplementary Figure 1

## Slide 2
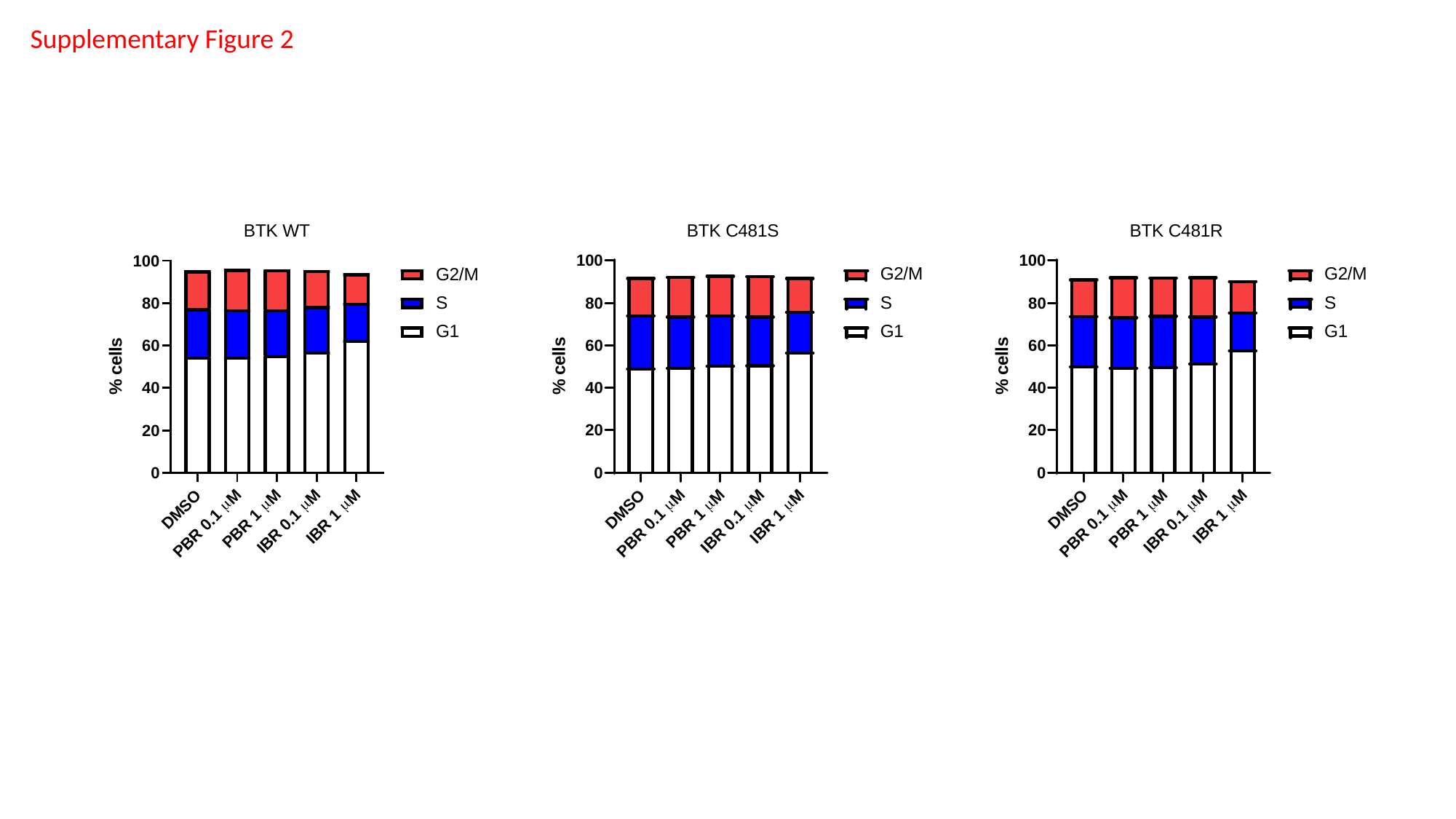

Supplementary Figure 2

## Slide 3
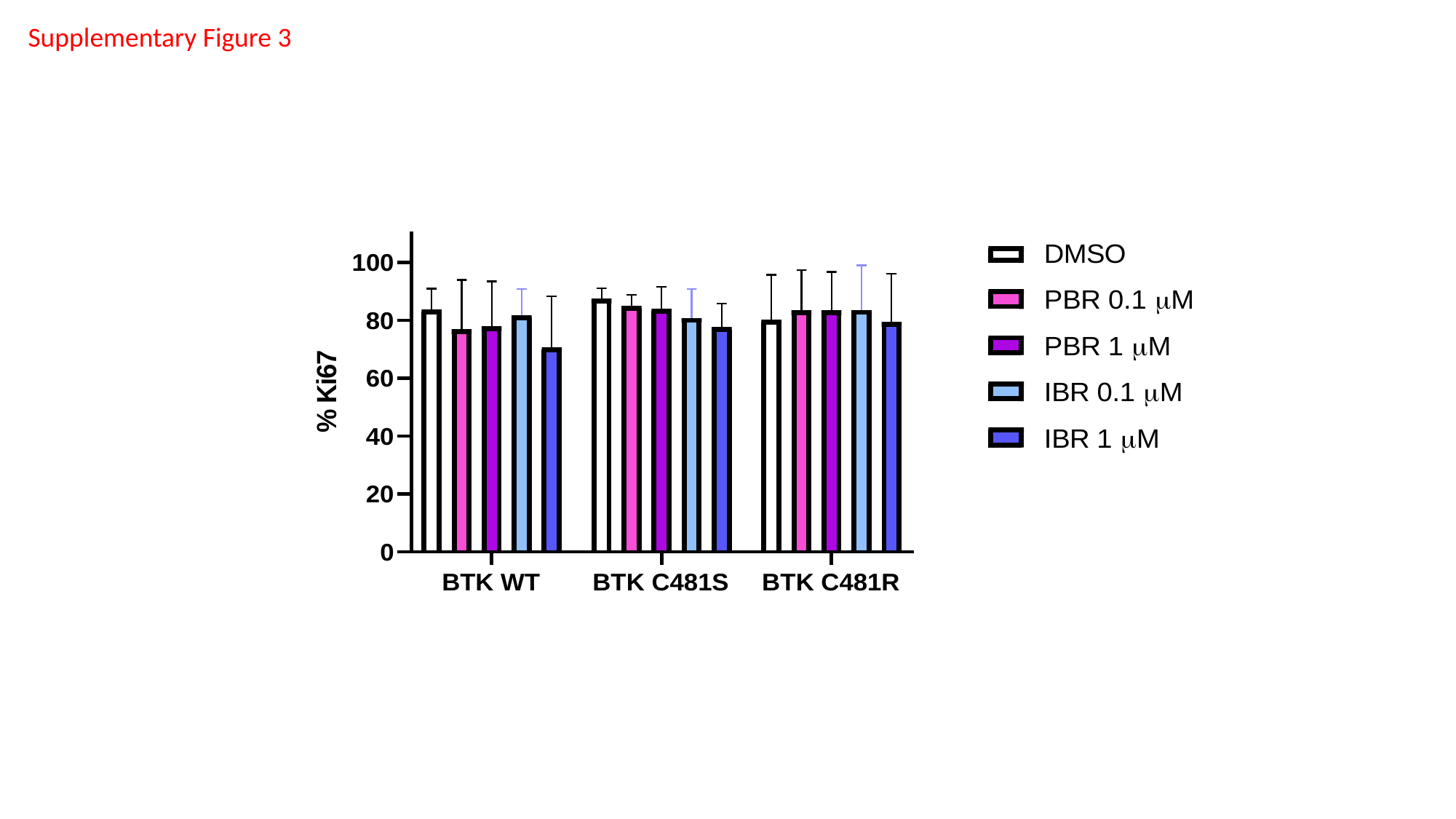

Supplementary Figure 3

## Slide 4
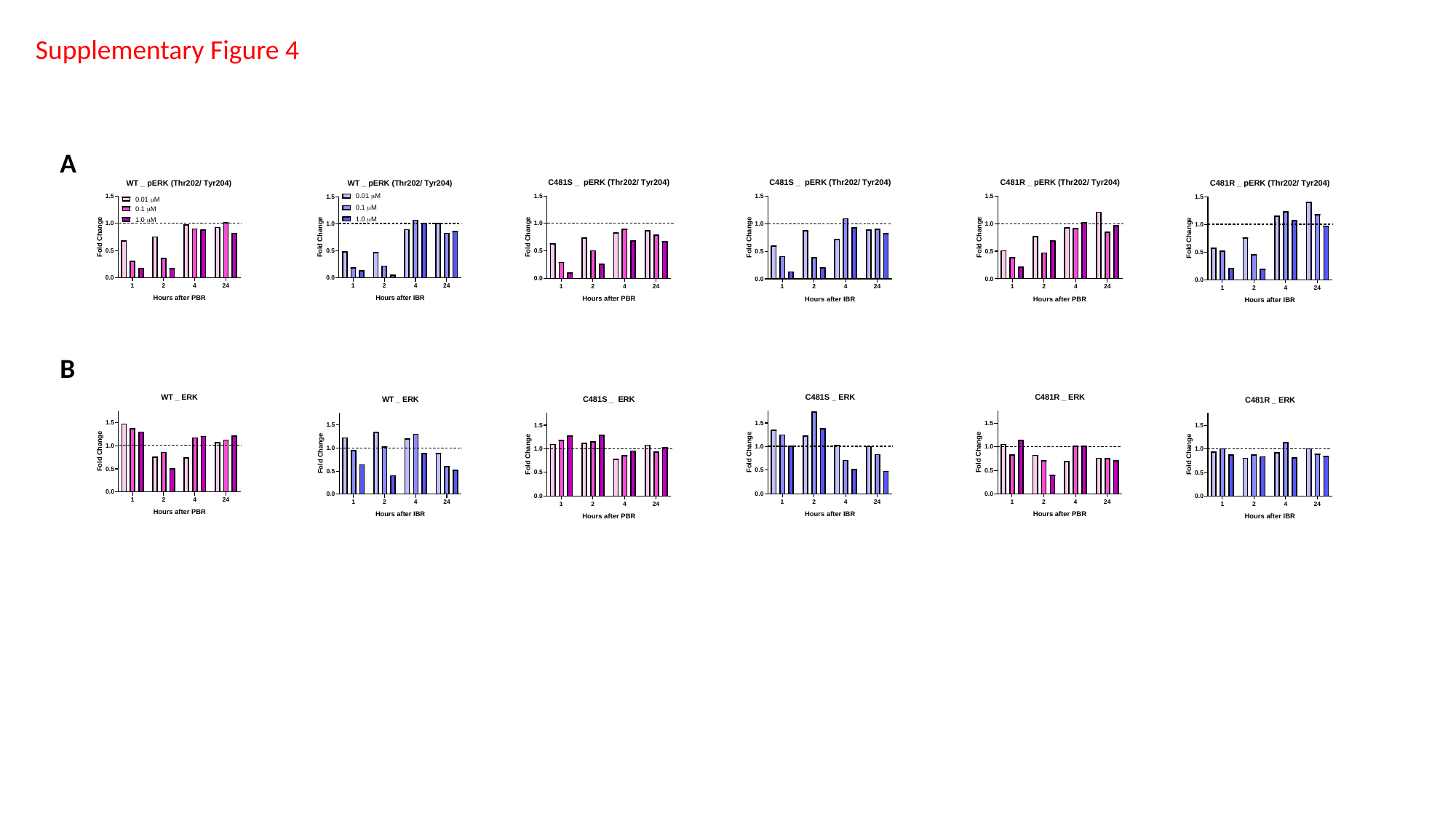

Supplementary Figure 4
A
B

## Slide 5
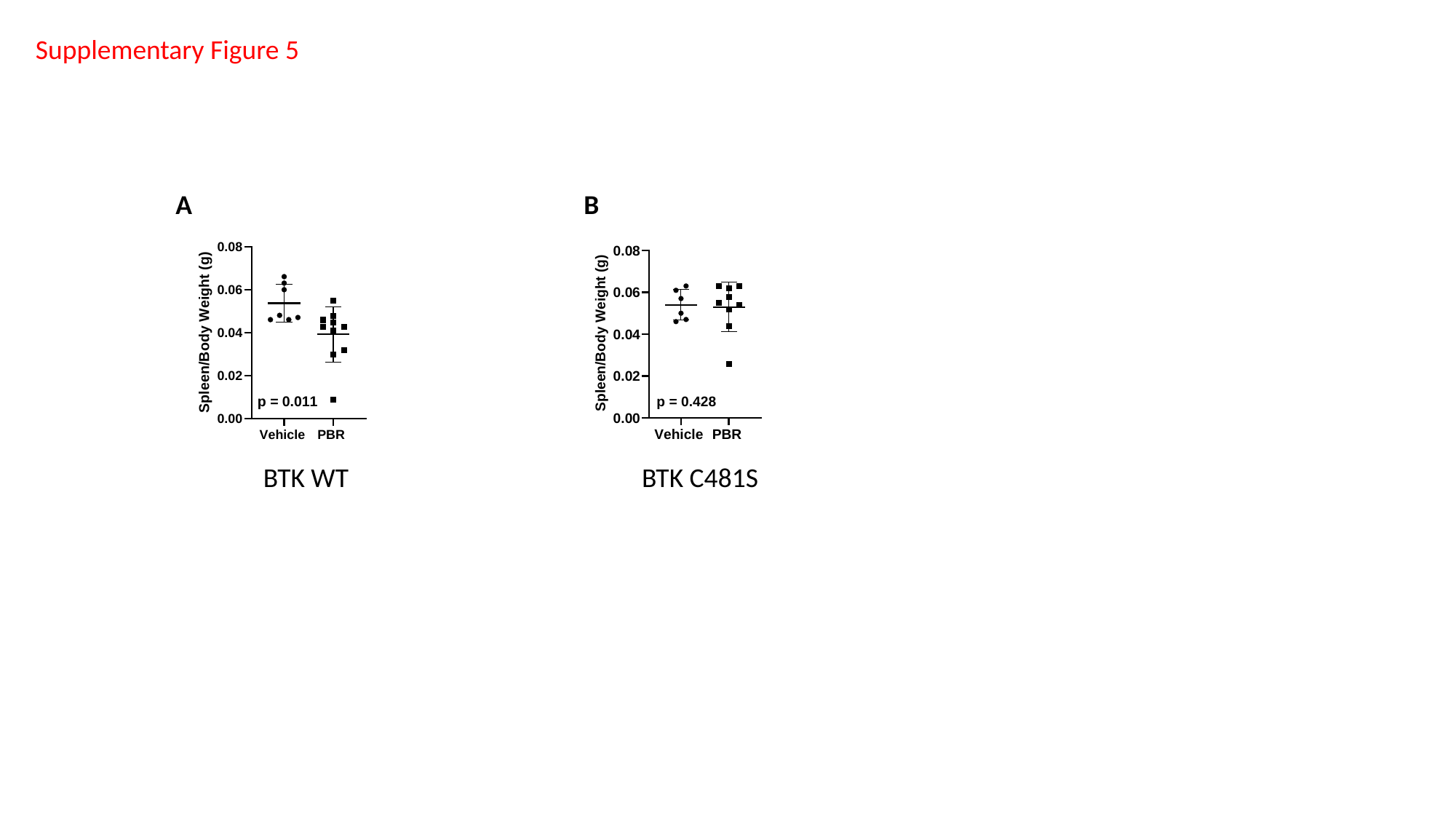

Supplementary Figure 5
A
B
BTK WT
BTK C481S

## Slide 6
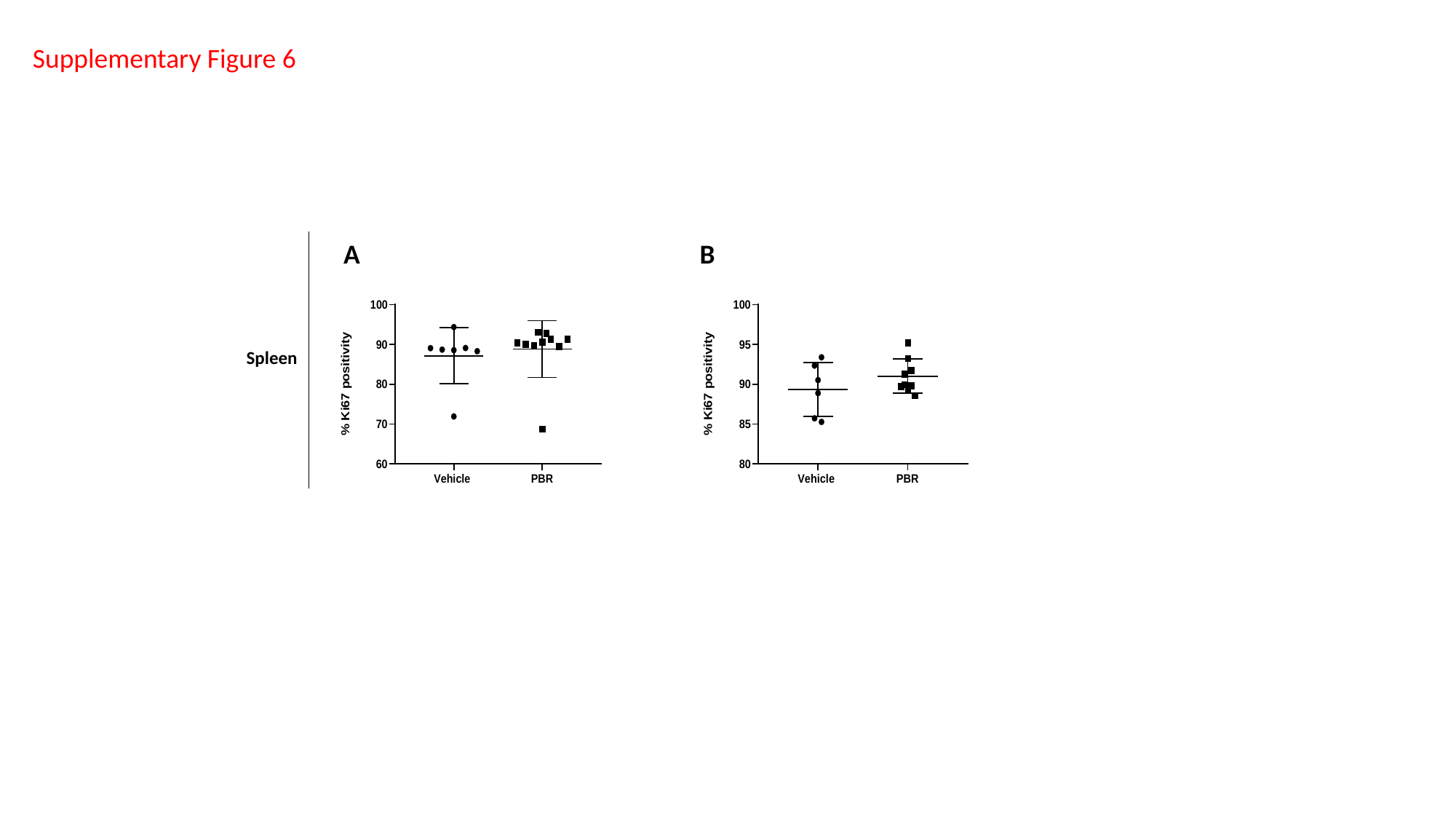

Supplementary Figure 6
A
B
Spleen

## Slide 7
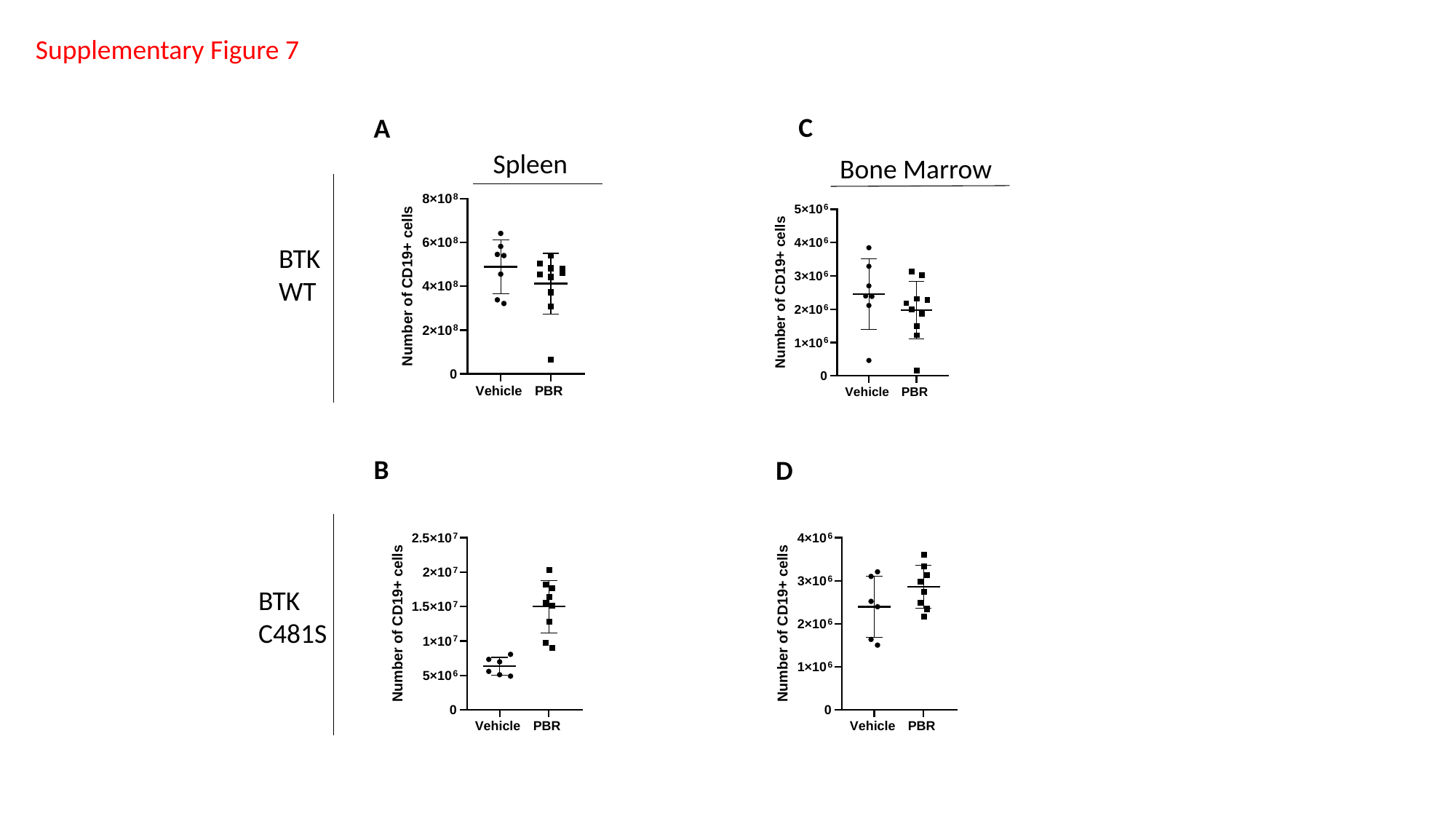

Supplementary Figure 7
C
A
Spleen
Bone Marrow
BTK
WT
B
D
BTK C481S

## Slide 8
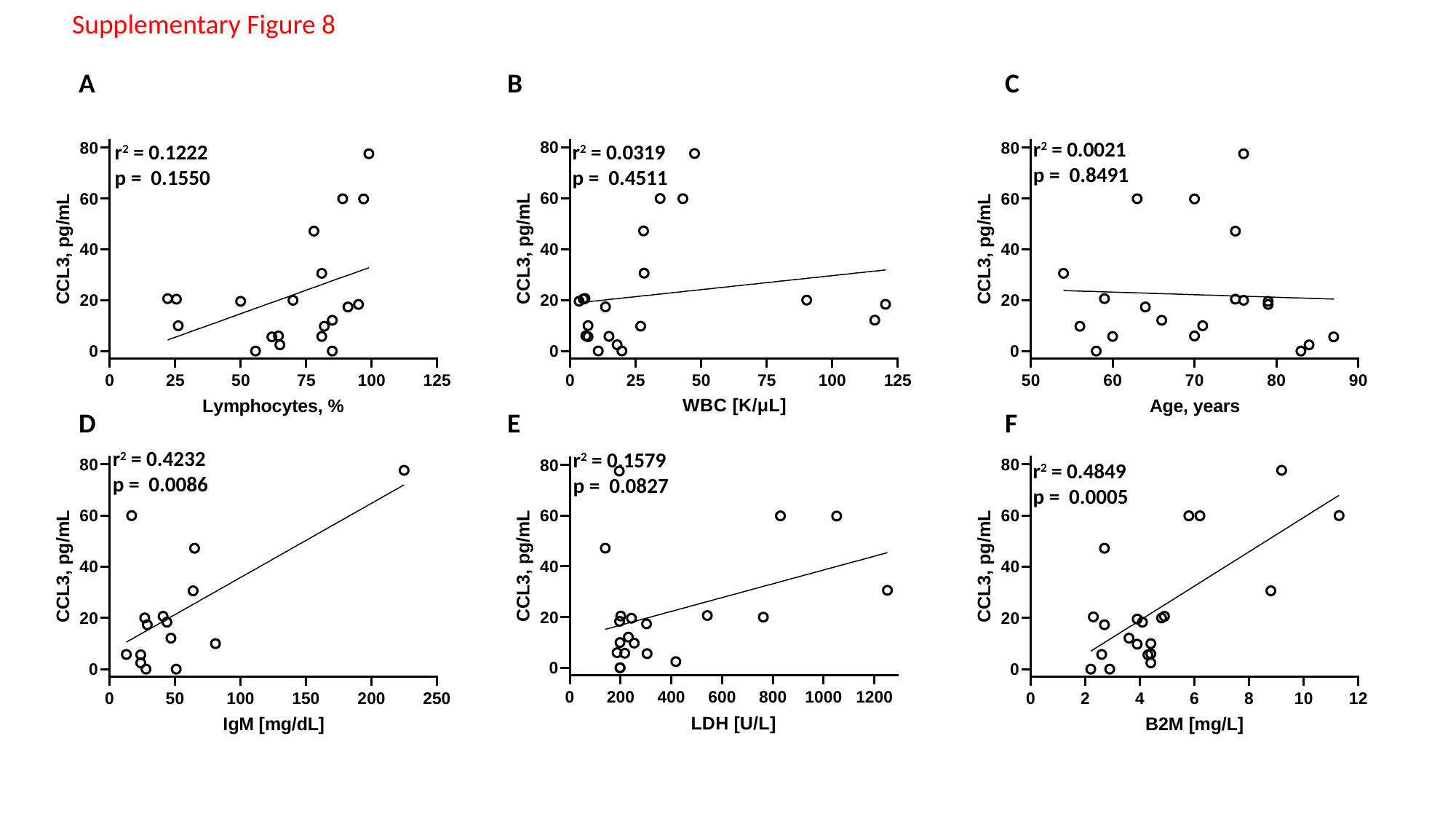

Supplementary Figure 8
A
B
C
r2 = 0.0021
p = 0.8491
r2 = 0.1222
p = 0.1550
r2 = 0.0319
p = 0.4511
D
E
F
r2 = 0.4232
p = 0.0086
r2 = 0.1579
p = 0.0827
r2 = 0.4849
p = 0.0005

## Slide 9
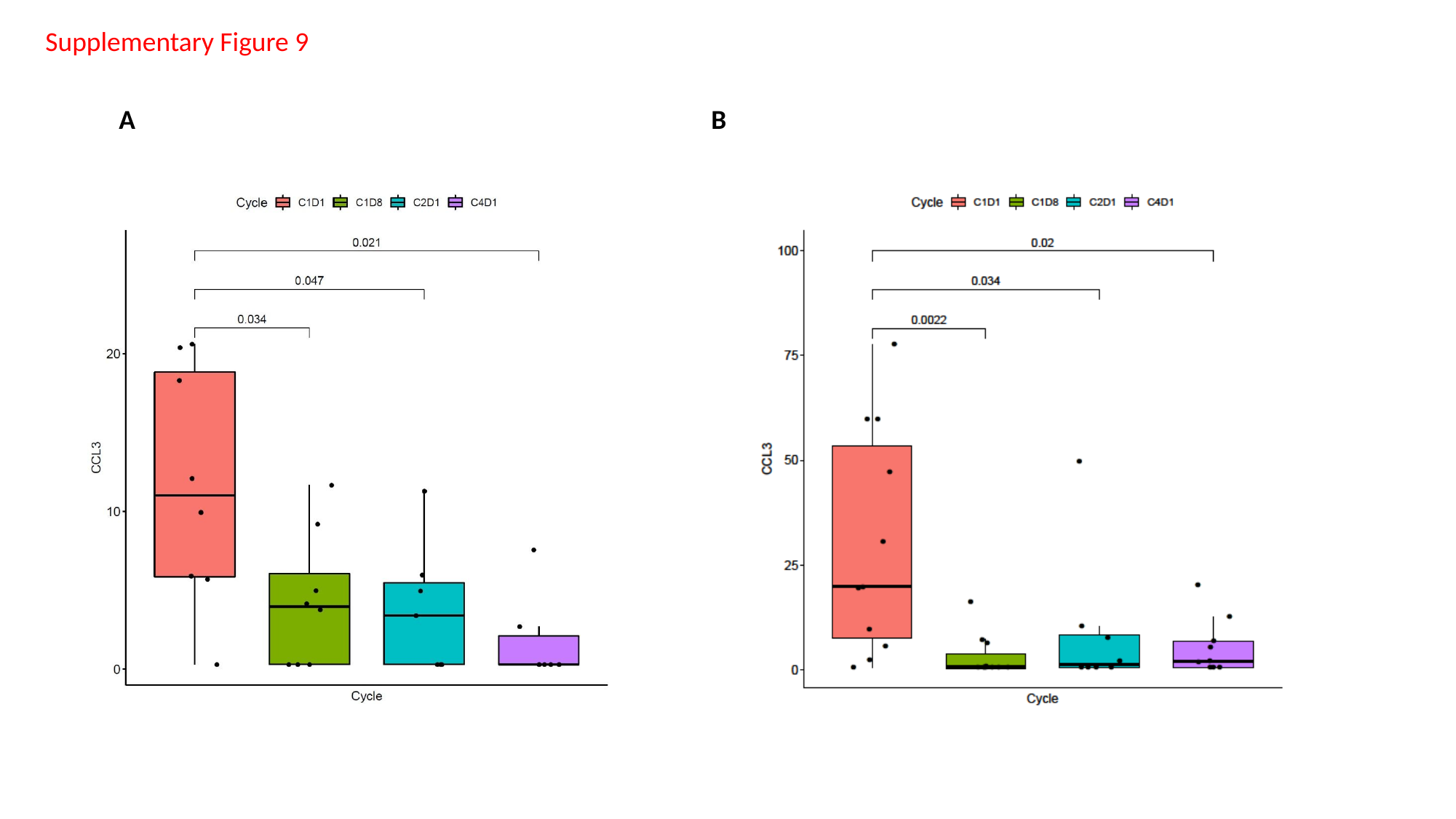

Supplementary Figure 9
A
B

## Slide 10
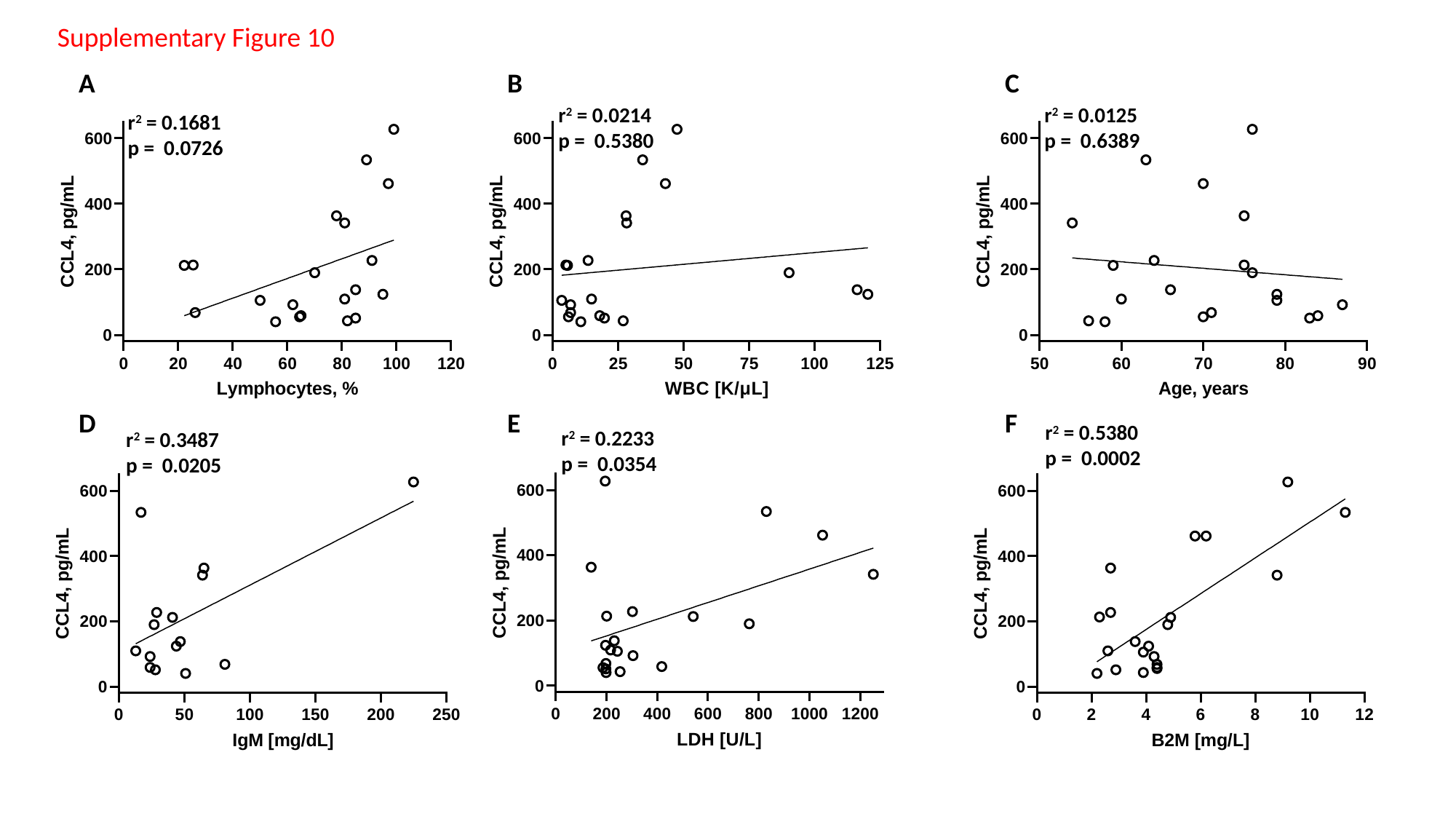

Supplementary Figure 10
A
B
C
r2 = 0.0214
p = 0.5380
r2 = 0.0125
p = 0.6389
r2 = 0.1681
p = 0.0726
D
E
F
r2 = 0.5380
p = 0.0002
r2 = 0.2233
p = 0.0354
r2 = 0.3487
p = 0.0205

## Slide 11
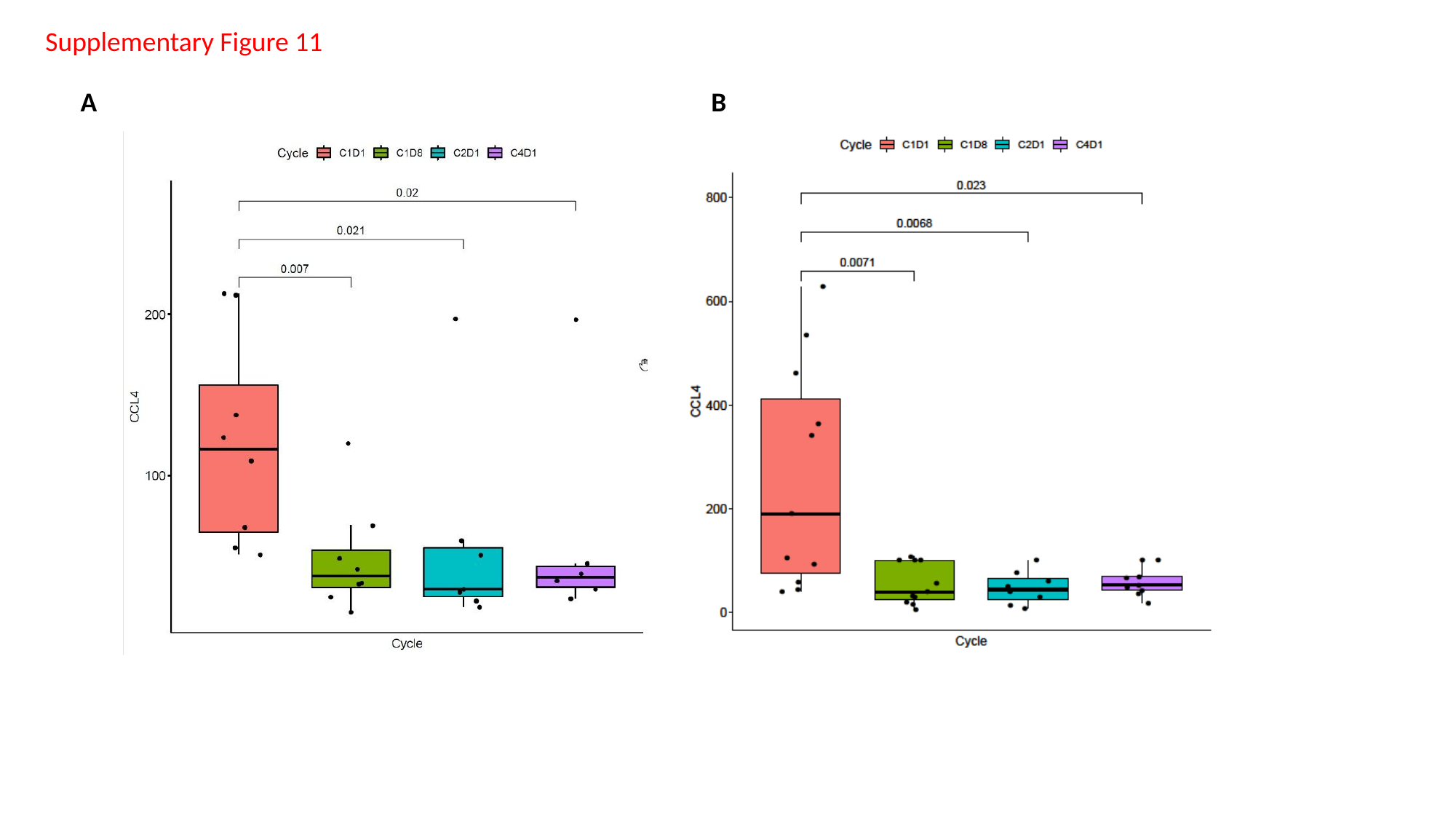

Supplementary Figure 11
A
B

## Slide 12
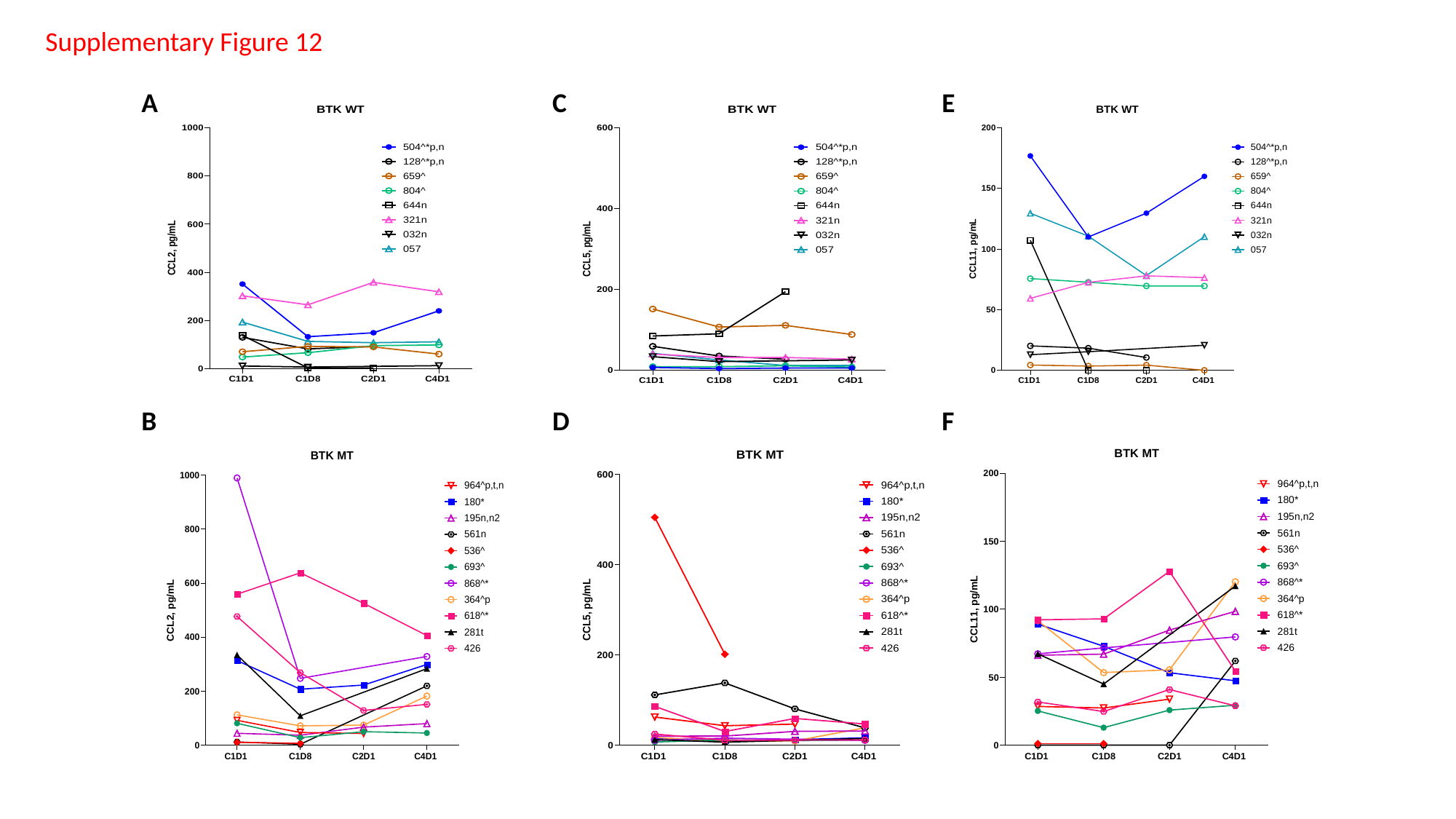

Supplementary Figure 12
A
C
E
B
D
F
