# Supplementary material for: Pirtobrutinib inhibits wild-type and mutant Bruton’s tyrosine kinase-mediated signaling in chronic lymphocytic leukemia
Source: Blood Cancer J. 2022 May 20;12(5):80. doi: 10.1038/s41408-022-00675-9 (PMC9123190; doi:10.1038/s41408-022-00675-9)
Supplement: Supplementary file 3 — Supplementary Figure Legends [file 41408_2022_675_MOESM3_ESM.docx]

**Supplemental Figure Legends**

**Supplemental Figure 1. Effects of pirtobrutinib or ibrutinib incubations on cell death of MEC-1 cells that overexpress wild-type or mutant BTK**.

Exponentially growing MEC-1 cells overexpressing wild-type or mutant variants of BTK were incubated with DMSO control or indicated concentrations of drugs for 24 hours. Cells were stained with annexin/propidium iodide (PI) as detailed in the methods. The percentage of annexin V–positive cells was determined by flow cytometry (BD Accuri C6, BD Biosciences) using BD Accuri C6 software (n=3).

**Supplemental Figure 2. Effects of pirtobrutinib or ibrutinib incubations on cell cycle profile of MEC-1 cells that overexpress wild-type or mutant BTK**.

Exponentially growing MEC-1 cells overexpressing wild-type or mutant variants of BTK were incubated with DMSO control or indicated concentrations of drugs for 24 hours. Treated cells were fixed in 70% ethanol and stained with PI, and cell cycle profiles were analyzed by flow cytometry (n=3).

**Supplemental Figure 3. Effects of pirtobrutinib or ibrutinib incubations on proliferation of MEC-1 cells that overexpress wild-type or mutant BTK**.

Exponentially growing MEC-1 cells overexpressing wild-type or mutant variants of BTK were incubated with DMSO control or indicated concentrations of drugs for 24 hours. Cells were stained with PE-labeled Ki67 antibody for proliferation. The percentage of proliferative cells (Ki67+) was determined by flow cytometry (BD Accuri C6, BD Biosciences) using BD Accuri C6 software (n=3).

**Supplemental Figure 4**. **Impact of pirtobrutinib or ibrutinib on ERK activity in MEC-1 cells harboring either BTK^WT^ or BTK^C481S^**.

**(A-B)** Dose and time-dependent inhibition of ERK phosphorylation after treatment with pirtobrutinib (pink bars) or ibrutinib (blue bars) in BTK^WT^, BTK^C481S^, and BTK^C481R^ variants of BTK overexpressing MEC-1 cells. Cells were treated at 3 different concentrations of drug at 4 different time points. Densitometry values are expressed as percentage of DMSO control.

**Supplemental Figure 5. Impact of pirtobrutinib on spleen/body weight in murine models expressing MEC-1 cells harboring either BTK^WT^ or BTK^C481S^**.

MEC-1 cells overexpressing BTK^WT^ or mutant BTK (1x10^7^ cells/mouse) were injected into 8-week-old Rag2^−/−^γ_c_^−/−^ female mice and the animals were monitored daily. At day 10, mice were randomly assigned to groups: vehicle (n=7 or n=6 in BTK^WT^ and BTK^C481S^ models, respectively) and pirtobrutinib (n=10, in both models), and treatment started. The experiment was terminated at day 26. At the endpoint, mice were euthanized and spleens were collected. Spleen/body weight ratios **(A)** in BTK^WT^ model (n=7 for vehicle, n=10 for pirtobrutinib) **(B)** in BTK^C481S^ model (n=6 for vehicle, n=10 for pirtobrutinib).

**Supplemental Figure 6. Impact of pirtobrutinib on number of Ki67-positive cells in spleen of murine models expressing MEC-1 cells harboring either BTK^WT^ or BTK^C481S^.**

MEC-1 cells overexpressing BTK^WT^ or mutant BTK (1x10^7^ cells/mouse) were injected into 8-week-old Rag2^−/−^γ_c_^−/−^ female mice and the animals were monitored daily. At day 10, mice were randomly assigned to groups: vehicle (n=7 or n=6 in BTK^WT^ and BTK^C481S^ models, respectively) and pirtobrutinib (n=10, in both models), and treatment started. The experiment was terminated at day 26. At the endpoint, mice were euthanized and spleens were collected. Cells were isolated from spleen and stained with Ki67 antibody followed by flow cytometry analysis. Percentage of Ki67 positivity in spleens **(A)** in BTK^WT^ model (n=7 for vehicle, n=10 for pirtobrutinib) **(B)** in BTK^C481S^ model (n=6 for vehicle, n=10 for pirtobrutinib).

**Supplemental Figure 7**. **Impact of pirtobrutinib on number of CD19+ cells in spleen and bone marrow in murine models expressing MEC-1 cells harboring either BTK^WT^ or BTK^C481S^**.

MEC-1 cells overexpressing BTK^WT^ or mutant BTK (1x10^7^ cells/mouse) were injected into 8-week-old Rag2^−/−^γ_c_^−/−^ female mice and the animals were monitored daily. At day 10, mice were randomly assigned to groups: vehicle (n=7 or n=6 in BTK^WT^ and BTK^C481S^ models, respectively) and pirtobrutinib (n=10, in both models), and treatment started. The experiment was terminated at day 26. At the endpoint, mice were euthanized and spleens and left femurs were collected. Cells were isolated from spleen and bone marrow and stained with a monoclonal antibody against PE-labeled human CD19 Clone J3119 (Beckman Coulter) followed by flow cytometry analysis. **(A** and **B)** Number of CD19+ cells in spleen and (**C** and **D)** number of CD19+ cells in bone marrow of mice with either BTK^WT^ **(A** and **C)** or BTK^C481S^ **(B** and **D)** harboring MEC-1 cells.

**Supplemental Figure 8. Relationship between CCL3 and other parameters in patients with CLL.**

Peripheral blood samples were collected prior to pirtobrutinib therapy. Patient samples here and in the following supplemental figures are from patients enrolled in a study registered at ClinicalTrials.gov (identifier NCT03740529). Plasma was collected at indicated timepoints and used for chemokine assays. CCL3 levels were quantitated using Luminex XMap Technology as described under Methods. Study included 20 patients. **(A)** Correlation of CCL3 and percentage of lymphocytes at zero time point during pirtobrutinib therapy (n=20; r^2^=0.12; p=0.16). **(B)** Correlation of CCL3 and white blood cell counts (K/µL) at zero time point during pirtobrutinib therapy (n=20; r^2^=0.03; p=0.45). **(C)** Correlation of CCL3 and age at zero time point during pirtobrutinib therapy (n=20; r^2^=0.002; p=0.85). **(D)** Correlation of CCL3 and IgM (mg/dL) at zero time point during pirtobrutinib therapy (n=20; r^2^=0.42; p=0.009). **(E)** Correlation of CCL3 and LDH (U/L) at zero time point during pirtobrutinib therapy (n=20; r^2^=0.16; p=0.08). **(F)** Correlation of CCL3 and B2M (mg/L) at zero time point during pirtobrutinib therapy (n=20; r^2^=0.49; p=0.0005).

**Supplemental Figure 9. Inhibition of CCL3 in CLL patients during pirtobrutinib therapy**

This study is registered at ClinicalTrials.gov (identifier NCT03740529). Plasma was collected at indicated time points and used for chemokine assays. Peripheral blood samples were collected prior to therapy (C1D1), 1 week (C1D8), 4 weeks or one cycle (C2D1), and three cycles (C4D1) after the start of pirtobrutinib. CCL3 levels were quantitated using Luminex XMap Technology as described under Methods. CCL3 levels are presented in **(A)** patients harboring BTK^WT^ (n=8) and **(B)** patients harboring mutant BTK (n=11).

**Supplemental Figure 10. Relationship between CCL4 and other parameters in patients with CLL.**

Peripheral blood samples were collected prior to pirtobrutinib therapy. This study is registered at ClinicalTrials.gov (identifier NCT03740529). Plasma was collected at indicated timepoints and used for chemokine assays. CCL3 levels were quantitated using Luminex XMap Technology as described under Methods. Study included 20 patients. **(A)** Correlation of CCL4 and percentage of lymphocytes at zero time point during pirtobrutinib therapy (n=20; r^2^=0.17; p= 0.07). **(B)** Correlation of CCL4 and white blood cell counts (K/µL) at zero time point during pirtobrutinib therapy (n=20; r^2^=0.02; p=0.54). **(C)** Correlation of CCL4 and age at zero time point during pirtobrutinib therapy (n=20; r^2^=0.013; p=0.64). **(D)** Correlation of CCL4 and IgM (mg/dL) at zero time point during pirtobrutinib therapy (n=20; r^2^=0.35; p=0.02). **(E)** Correlation of CCL4 and LDH (U/L) at zero time point during pirtobrutinib therapy (n=20; r^2^=0.22; p=0.04). **(F)** Correlation of CCL4 and B2M (mg/L) at zero time point during pirtobrutinib therapy (n=20; r^2^=0.54; p=0.0002).

**Supplemental Figure 11. Inhibition of CCL4 during pirtobrutinib therapy in CLL patients harboring mutant BTK.**

This study is registered at ClinicalTrials.gov (identifier NCT03740529). Plasma was collected at indicated time points and used for chemokine assays. Peripheral blood samples were collected prior to therapy (C1D1), 1 week (C1D8), 4 weeks or one cycle (C2D1), and three cycles (C4D1) after the start of pirtobrutinib. CCL4 levels were quantitated using Luminex XMap Technology as described under Methods. CCL4 levels are presented in **(A)** patients harboring BTK^WT^ (n=8) and **(B)** patients harboring mutant BTK (n=11).

**Supplemental Figure 12. Inhibition of chemokine production during pirtobrutinib therapy in CLL patients previously treated with irreversible BTKi.**

Peripheral blood samples were collected prior to therapy (C1D1), 1 week (C1D8), 4 weeks or one cycle (C2D1), and three cycles (C4D1) after the start of pirtobrutinib. This study is registered at Clinicaltrials.Gov (identifier NCT03740529). Plasma was collected at indicated time points and used for chemokine assays. CCL2 (MCP-1) **(A-B),** CCL5 (RANTES) **(C-D)** and CCL11 (Eotaxin) **(E-F)** levels were quantitated using Luminex XMap Technology as described under Methods. Study included patients with BTK^WT^ (n=8) and mutant BTK (n=12). BTK double kinase mutants are depicted by solid squares (patients 618 [BTK^C481S+C481R^] and 180 [BTK^C481F+C481S^]). BTK kinase domain as well as gate-keeper mutants are indicated by hexagon with dot (patients 426 [BTK^C481S+T474^] and 561 [BTK^C481S+T474^]). Patient 845 received pirtobrutinib and venetoclax combination and was not included in the time course; patients 364 and 128 had Richter transformation. Patients’ additional mutations are indicated in the key by a caret (TP53), navy blue color (BCL2), n (NOTCH1), n2 (NOTCH2), and asterisk (PLCG2), p indicates 17pdel and t indicates trisomy 12.
