# Supplementary material for: Pirtobrutinib inhibits wild-type and mutant Bruton’s tyrosine kinase-mediated signaling in chronic lymphocytic leukemia
Source: Blood Cancer J. 2022 May 20;12(5):80. doi: 10.1038/s41408-022-00675-9 (PMC9123190; doi:10.1038/s41408-022-00675-9)
Supplement: Supplementary file 4 — Supplementary Tables [file 41408_2022_675_MOESM4_ESM.docx]

**Supplementary Table 1. Patient information about BTK and other mutations**

| **Pt #** | **BTK status** | **BTK**  **%VAF** | **Other mutations** |
| --- | --- | --- | --- |
| 644 | WT |  | ATM, NOTCH1, FAT1 |
| 321 | WT |  | MYC, ATM, STK11, ZRSR2, NOTCH1, ZFAT, SPEN, PLEKHG5, POLE, PRDM1, KMT2D, BCL10, FAT1 |
| 032 | WT |  | NOTCH1 |
| 504 | WT |  | TP53, SPEN, NOTCH1, TET2, BCL2, SPEN, SETD2, IGLL5, PLCG2 |
| 659 | WT |  | TP53, ASXL1, BRAF, FAT1, MYC, NF1, PAX5, PLEKHG5, RB1, SOX11, |
| 804 | WT |  | TP53 |
| 057 | WT |  | Not available |
| 128 | WT |  | ID3, TP53, FAT1, ATM, MAPK1, NOTCH1, POLE, PRDM1, RBMX, ZFAT, PLCG2 |
| 116 | WT |  | BCL2, RPS15, SF3B1, TET2, TP53, SP140, HIST1H1C, PLEKHG5 |
| 845 | C481S | 30 | IGLL5, TP53, CIITA, FAT1, FOXO1, H1-2, H1-2, KMT2D, PRDM1, TCF3, TENT5C, TET2 |
| 536 | C481S | <2 | FAT1, MUC2, ZMYM3, TP53 |
| 693 | C481S | 86 | TP53, MYC, SPEN, PLEKHG5, TET2, KMT2D, CIITA, CNOT3 |
| 281 | C481S | 63 | CXCR4, XPO1, FAT1 |
| 195 | C481S | 13 | NOTCH1, SAMHD1, TET2, ATM, KMT2D, NOTCH2, RBMX, TET2, ZFAT |
| 964 | C481S | 6 | DNMT3A, TP53, ATM, HVCN1, KMT2D, MED12, SPEN, BAZ2A, IRAK1, NOTCH1, PTPN1, RFTN1, SETD2, TENT5C, TNFAIP3 |
| 868 | C481R | <5 | TP53, ASXL1, SF3B1, FAT1, KMT2D, PLCG2, RBMX, ZFAT |
| 364 | C481S | NA | TP53, MUC2 |
| 618 | C481S | <5 | CIITA, FAT1, H1-2, KMT2D, NFKBIA, PLCG2, PTPN1, RBMX, S1PR1, STAT3, STK11, TNFAIP3, TP53, ZRSR2 |
|  | C481R | 18 |  |
| 561 | C481S | 20 | XPO1, SPEN, NOTCH1 |
|  | T474I | 20 |  |
| 180 | C481F | 23 | BCL2, SF3B1, FAT1, IGLL5, KMT2D, MYC, PLCG2, PLEKHG5, TNFAIP3 |
|  | C481S | 18 |  |
| 426 | C481S | <5 | XPO1, ARID1A, POLE, TET2, ATM, ASXL1, NFKBIE, ELF4, CNOT3, JAK2, KMT2D, FAM46C, MAPK1, RBMX, TNFAIP3, NXF1 |
|  | T474I | <5 |  |

| BTK, Bruton Tyrosine Kinase; NA, Not available; WT, BTK wild type; VAF, Variant Allele Frequency |
| --- |

**Supplementary Table 2. List of antibodies used**

| **Protein** | **kDa** | **Antibody Name** | **Supplier** | **Catalog No** |
| --- | --- | --- | --- | --- |
| Vinculin | 124 | Vinculin (E1E9V) XP | Cell Signaling | 13901 |
| BTK | 77 | Purified Mouse Anti-Human Btk  Clone  53/BTK  (RUO) | BD Biosciences | 611116 |
| pBTK | 77 | Phospho-Btk (Tyr223) Antibody | Cell Signaling | 5082 |
| p-S6 | 32 | Phospho-S6 Ribosomal Protein (Ser235/236) | Cell Signaling | 4858 |
| S6 | 32 | S6 Ribosomal Protein (54D2) | Cell Signaling | 2317 |
| p-PLCɣ2 | 150 | Phospho-PLCγ2 (Tyr1217) | Cell Signaling | 3871 |
| PLCɣ2 | 150 | PLC γ2 Antibody (B-10) | Santa Cruz | Sc-5283 |
| p-ERK | 44,42 | Phospho-p44/42 MAPK (T202/Y204) (D13.14.4E) | Cell Signaling | 4370S |
| ERK | 44,42 | P44/42 MAPK (ERK 1/ 2) (3A7) | Cell Signaling | 9107 |
| Bcl-2 | 26 | BCL-2 Antibody | Dako | M0887 |
| Puma | 23 | Puma Antibody | Cell Signaling | 4976 |
| p-AKT | 60 | Phospho-AKT (Ser473) Antibody | Cell Signaling | 9271 |
| AKT | 60 | AKT Antibody | BD Biosciences | 610817 |
| PARP | 89,116 | PARP | BD Biosciences | 556362 |
| p-NFKB | 65 | Phospho- NF-κB p65 (Ser536) | Cell Signaling | 3031 |
| NFKB | 65 | NF-κB p65 (L8F6) | Cell Signaling | 6956 |
| Mcl-1 | 40 | Mcl-1 Antibody | Invitrogen | AHO0102 |
| Bcl-XL | 30 | Bcl-XL Antibody | Cell Signaling | 2762 |
| Bim | 12,15, 23 | Bim (Rabbit) Cell Signaling | Cell Signaling | 2933 |
| Bax | 20 | Bax Antibody | Sigma | B8554 |
